# Electrically Switchable Molecular Adhesion via Self-Assembled Monolayer-Mediated Hydration and Ion Structuring

**DOI:** 10.1021/jacs.5c11903

**Published:** 2025-11-07

**Authors:** Valentina Wieser, Yoyo Cheng-Ting Yu, Andrea Valencia Ramirez, David T. Wu, Frank Uwe Renner, Hsiu-Wei Cheng

**Affiliations:** † Department of Chemistry, 33561 National Taiwan University, Taipei 10617, Taiwan; ‡ Institute of Chemistry, 71552 Academia Sinica, Taipei 115, Taiwan; § Sustainable Chemical Science and Technology, Taiwan International Graduate Program, Academia Sinica and National Taiwan University, Taipei 10617, Taiwan; ∥ Department of Chemical Engineering, 33561 National Taiwan University, Taipei 10617, Taiwan; ⊥ Institute for Materials Research (IMO), 54496 Hasselt University, Wetenschapspark 1, 3590 Diepenbeek, Belgium; # IMEC vzw. Division IMOMEC, 3590 Diepenbeek, Belgium; ∇ 31276 Competence Center for Electrochemical Surface Technologies CEST, Viktor-Kaplan-Strasse 2, 2700 Wiener Neustadt, Austria; ○ Institute of Applied Physics, TU Wien, Wiedner Hauptstrasse 8-10/E134, 1040 Wien, Austria

## Abstract

The interplay of specific surface interactions as well as ion and hydration structuring takes on a pivotal role in dictating the intermolecular, intersurface, and colloidal behavior at solid–liquid interfaces. The detailed atomic and molecular structure consequently influences a wide array of surface-mediated functions in technological and biological systems. Ion and hydration structuring at the interface is susceptible to various surface parameters, including surface potential, structural modifications including molecular adsorbents, the charge of specific functional groups, and electrolyte composition. Here, we disclose an electromechanical adhesion switch mechanism and demonstrate, in operation, the impact of molecular surface modification and potential modulation on adhesive and repulsive forces between surfaces. We exemplify these fundamental interactions by measuring the acting intermolecular forces between mica and metal surfaces modified with self-assembled monolayers including mercaptobenzimidazole and cysteamine films, showcasing the potential for tailoring surface interactions via ion adsorption manipulation. Employing an electrochemical surface forces apparatus complemented with molecular dynamics simulation, we present a comprehensive analysis of the specific forces involved in film–mica interactions and the impact of ion ordering under electrochemical modulation on such forces. Our results offer a novel perspective on how hydration and ion adsorption shape solid–solid interactions involving organic thin films and how these interactions provide a flexible route for electromechanical adhesion switches.

## Graphical abstract

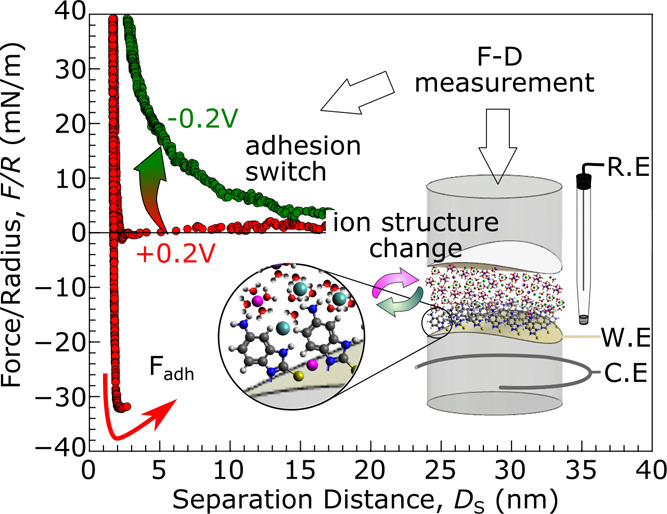

## Introduction

Interfacial interactions, often governed by electrochemical processes, are critically influenced by atomic and molecular structure, chemical composition, and reaction kinetics and play a central role across disciplines ranging from biology to material science and technology. Moreover, heterogeneous catalysis, corrosion, battery interfaces, and many biological systems consist of at least one electrochemical interface. On the other hand, adhesion, as a driving interaction force between two surfaces, is important to describe particle agglomeration of battery active materials, the design of detachable adhesives,^1^ (bio) lubrication, biomimetic glues,^2,3^ or robotic^4^ and AFM grippers,^5^ all the way to understanding stress corrosion cracking^6^ or fin collapse in nanoscale semiconductor etching.^7^

Adhesion forces in electrochemical systems are accordingly influenced by the surface chemical structure, including functionalization, e.g., by organic adsorbent layers, and the electric double layer (EDL), where ion adsorption and near-surface hydration are key features.^8–10^ With the interplay of electrostatic forces, dipole, and van der Waals interactions, so-called structural forces in water, such as hydration and hydrophobic forces,^11,12^ adhesion relates to a complex multidimensional energy landscape. Indeed, tailored interactions within the EDL between functional groups at the surface and the surrounding electrolyte solution are often what drive vital specific surface interactions. While controlling the specific access and blocking of other reactants through steric or electronic effects at the interface is desired in electrocatalysis applications,^13,14^ effective lubrication of surfaces via specific ion and water trapping is a welcome effect in biolubrication.^15^

From the classical point of view, the EDL forms a structured ionic environment at the interface (‘ion storage’) that is divided into the inner and outer Helmholtz planes or Stern layer, a layer of densely packed counterions directly physisorbed at the interface, and a more mobile layer of solvated counterions in the diffuse layer, which screens the rest of the surface charge according to a Poisson–Boltzmann distribution.^16^ However, this EDL model and its interaction force description, encompassed in the Derjaguin–Landau–Verwey–Overbeek (DLVO) theory, are limited to systems fulfilling particular boundary conditions. The DLVO model, for example, considers only Coulombic interactions between a flat surface and point charges. Therefore, predictions fail when the system exhibits non-charge–charge interaction, e.g. structural forces such as hydrophobic interaction and hydration-related forces, or when the ion concentration at the interface is too high for the ion size to be negligible.^10,17^

In recent years, both experimental and theoretical understanding of surface structures and adhesion layers, as well as ion and hydration structuring within the innermost layers of the EDL, have advanced significantly. This progress owes much to the application of cutting-edge techniques using synchrotron light^18^ or scanning probe techniques such as atomic force microscopy (AFM),^19^ as well as spectroscopic tools such as sum frequency generation (SFG) spectroscopy.^20,21^ Furthermore, advanced simulation techniques have contributed at an increasing pace, helping to understand interface structuring on a deeper, molecular level.^22,23^ These methods have unveiled a more detailed view of ion and water structuring beyond the classical Gouy–Chapman–Stern model and underlined the general profound influence of ion structuring on overall surface interactions such as adhesion. However, a detailed understanding of near-interface ion and hydration structuring, particularly in the presence of organic adsorption layers, remains elusive, leaving open questions about their role in adhesion modulation.

One particularly prominent technique for capturing the complex intermolecular forces acting at the interface is the surface forces apparatus (SFA). This spectroscopic technique is based on interferometric distance measurement between two cross-cylindrically arranged, semitransparent mirrors and on force probing, enabling in situ measurement of intermolecular forces over large separations with high resolution.^24^ SFA can characterize interfaces with organic adsorbents such as self-assembled monolayers (SAMs)^25^ and, when combined with electrochemical tools (Figure 1a), allows in-operando investigation and tuning of EDL properties via a respective change of surface potentials.^26–28^

**Figure 1:**
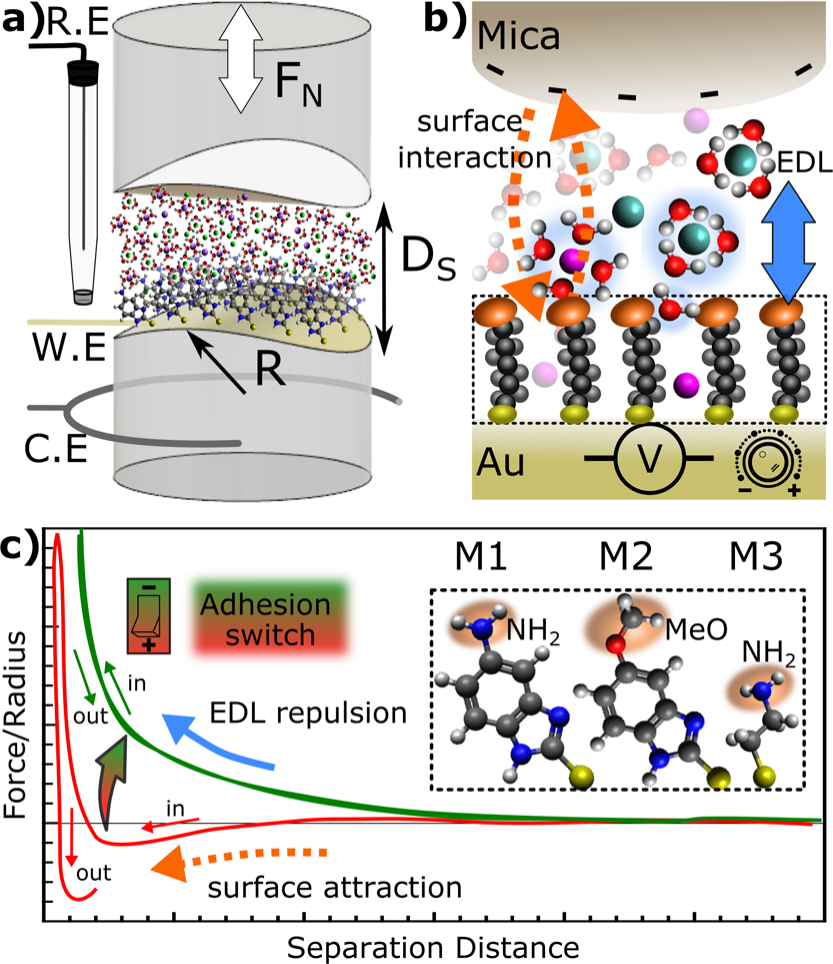
(a) Cross-cylindrical SFA setup consisting of a back-silvered mica surface apposing a gold electrode functionalized with a self-assembled monolayer, as well as a Pt wire as counter electrode and an Ag|AgCl reference electrode to complete the 3 electrode system. (b) Potential modulation of the gold electrode leads to different ion and water structuring within the innermost EDL, altering the overall interaction mechanism between the SAM and mica surface, effectively creating an electromechanical adhesion switch out of the SAM. (c) Exemplary force–distance measurement curve within the SFA geometry between mica and one of the 3 different SAM functionalizations specified in the inset, where M1 is 5-Amino-2-Mercaptobenzimidazole, M2 is 2-Mercapto-5-Methoxybenzimidazole, and M3 is Cysteamine. From the compression and separation profile, we can deduce either repulsive or adhesive interaction mechanisms.

Here, we use adlayer-modified surfaces within an electrochemical SFA setup (Figure 1) to measure and actively manipulate interaction forces between a variety of organic films and mica surfaces through external polarization. As examples, we chose mercaptobenzimidazole (SH-BimH) with amino (M1) and methoxy (M2) functional groups, as well as a linear cysteamine (M3) functionalization. With varying terminal functional groups (e.g., amine and methoxy), surface charge and adhesion forces can be fine-tuned. Such molecules form SAMs and have been studied, for example, in context of corrosion inhibitors^29,30^ or electrodeposition/biosensing platforms^31^ and also in the context of adhesion control.^32^

Additionally, ion adsorption and Stern layer hydration can impact adhesion mechanisms, tuning attractive and repulsive states.^33,34^ In this work, our electrochemical modulation enables us to control and describe, in particular, the specific cation and anion structuring in and at the organic thin film, effectively creating an electromechanical adhesion switch.

This phenomenon underscores the critical role of Stern layer hydration and ion ordering in interfacial interaction, achieving precise control without high potentials or complex redox chemistry.^3,35^ Our SFA measurements thus illuminate a pathway toward precise control and manipulation of intermolecular interactions at the solid–liquid interface solely utilizing EDL phenomena. Together, the presented results allow an advanced understanding of the complexity of interfaces and the interplay between near-surface hydration, ion adsorption, and adhesion mechanisms involving targeted surface functionalization. Our insights herald promising prospects for future applications in diverse scientific and technological fields.

## Results and Discussion

The SFA setup used in this work and schematically drawn in Figure 1a uses a highly sensitive strain gauge to measure the intermolecular forces acting between functionalized and polarizable gold and apposing mica surface. Combined with the interferometric distance measurement, we can then record force–distance (F–D) profiles.

For SH-BimH-5NH_2_ (M1) functionalized gold (WE) and mica surfaces in 1 mM NaCl, neutral pH solution, Figure 2 shows representative F–D measurements. The shown curves demonstrate the construction of an effective adhesion switch between attractive and repulsive behavior via application of positive and negative external polarizations against an Ag|AgCl reference, respectively.

**Figure 2:**
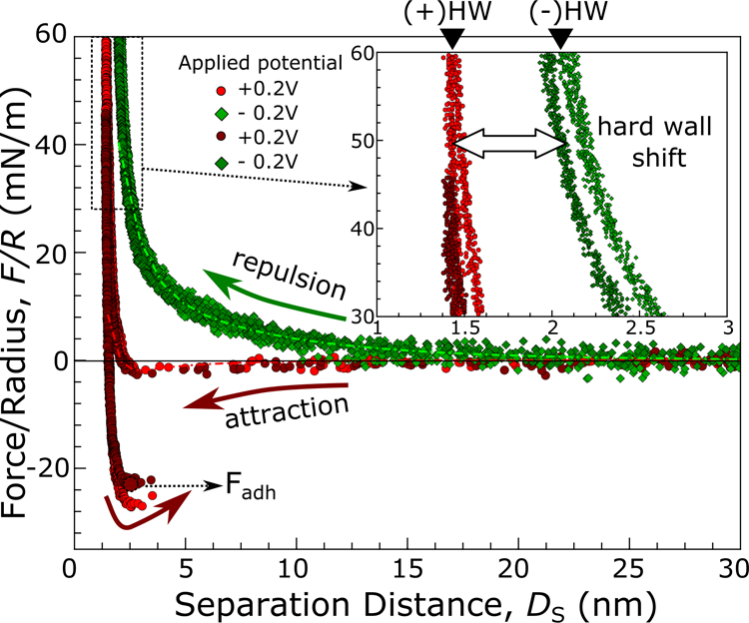
Force–distance (F–D) profiles upon compression and separation of a SH-BimH-5NH_2_ (M1) functionalized gold and mica surfaces at external polarizations of +0.2 and −0.2 V vs Ag|AgCl. Initial compression and separation at +0.2 V (light red circles) show attractive interaction with jump in and adhesion upon separation, whereas a subsequent F–D profile recorded at −0.2 V polarization (light green diamonds) exhibits purely repulsive behavior. Repeated compression and separation at +0.2 V (dark red circles) and −0.2 V (dark green diamonds) exhibit reversibility. DLVO fits are shown for both the +0.2 and −0.2 V compression curves as the red and green dash-dotted lines, respectively.

The observed F–D isotherms at an applied potential of +0.2 V (red circles) present a clear attractive behavior at separation distances below *D*_S_ ≈ 10 nm during the approach of the two surfaces as they are forced into an attractive minimum. As for separation, a very significant adhesive region with adhesion forces as high as −20 mN/m can be observed. On the other hand, polarization of −0.2 V (green diamonds) results in a fully repulsive compression and separation isotherm. This behavior is indeed reproducible over a large range of repetitions at a similar magnitude within the same experimental contact, as well as across other surface pairs. To show the reversibility of the switch character, we show measurements at the same contact position after 5–8 consecutive experiments, switching between +0.2 and −0.2 V external polarization in Figure 2 as dark red and green data, overlapping the respective previous isotherms at the same external polarization.

Beyond the stark difference in the measured adhesion behavior, we can also observe a very pronounced difference in the separation distance in regions of high compression force (see the inset graphic in Figure 2). In the region where *D*_S_ < 5 nm, the compression isotherm is mainly dominated by steric repulsion between SAM and mica, vdW attraction, and hydration repulsion. When an increase in compression force leads to a negligible change in separation distance, we reach the so-called “hard wall” (HW) as depicted in Figure 2. Comparing the cases of +0.2 and −0.2 V polarization, there is a clear outward shift in the HW distance (HW shift) of ∼9 Å for isotherms under cathodic polarization at compressive forces of ≥40 mN/m.

The pronounced repulsion, in addition to the associated buildup of an incompressible layer at negative potentials (HW shift), suggests the presence of a very structured cation and water layer, preventing an attractive interaction between SAM and mica.

Cation-driven hydration structuring due to electrostatic interaction between the surface charge and the ion and its effect on adhesion has been observed in bare mica-gold systems.^10,33,34,36,37^ Tivony et al. interpreted their results on cathodic polarization, favoring adsorption of cations at the interface of bare gold and mica surfaces due to the unique ability of cations to condense onto the surface and also keep tightly associated water trapped between surfaces of similar potential.^33^

In our work, the addition of specific surface functionalization in the form of the organic thin films now adds further complexity by contributing specific interaction with the mica as well as with the ions themselves, while showing unprecedented interaction stability. While it is known in biochemistry that organic layers such as phosphatidylcholine bilayers can exhibit guided hydration due to specific affinity toward Na^+^,^38,39^ the specific interaction between our selected, structurally much simpler, monolayers with mica and surrounding electrolyte results in high adhesive strength, with high contact stability and reproducibility.

To quantify the observed HW shift and adhesion switch behavior at negative polarizations and relate it to additional ion adsorption and accompanied hydration, we performed a DLVO fitting of the F–D curve with a model including a hydration repulsion term^38,40^ and charge regulation parameter.^41^ The mathematical detail and fitting parameter definition of the used DLVO theory are explained in the Supporting Information. Importantly, the DLVO fits for the curves in Figure 2, shown as green and red dash-dotted lines, reveal that indeed, the F–D profile at −0.2 V is largely dominated by hydration and EDL repulsion, considering the long hydration decay length λ_hyd_ = 0.9 nm as well as the high absolute gold surface potential ψ_Au_ = −0.07 V. In the case of positive polarization, the DLVO fit yields a significantly reduced hydration decay length λ_hyd_ = 0.35 nm as well as a more positive surface potential ψ_Au_ = −0.005 V. The results clearly correlate the hydration decay length λ_hyd_ with the applied potential *E*_vs Ag|AgCl_ and the surface potential ψ_Au_, indicating the hydration structure in such a SAM system can be electrochemically modulated. Furthermore, linking the near-surface hydration to the surface potential and hence the charge-regulating mechanism demonstrates that the hydration structure is ion correlated. Another evidence for ions driving the pronounced hydration structuring is given by reference measurements in Milli-Q water shown in Figure S2. Even though pure water measurements preserve the tuning between attractive and repulsive behavior for some individual measurements upon toggling of the external potential between positive and negative values, only a minor in comparison and not reproducible, HW shift is observed due to the lack of ions at the interface, aiding in the hydration layer organization. The fitted hydration decay length reflecting the buildup of an ordered ion/hydration layer does hence not show a clear surface polarization-dependent trend (Figure S3). While the EDL on the mica side can influence the sensed EDL structure on the gold, the slow displacement speed in SFA (≈3 nm/s) enables us to conduct equilibrium measurements and keep the influence of confinement effects imposed from the mica, albeit minimal, constant across the different surface polarizations of the gold. By tuning the degree of surface polarization and consequently the degree of ion and hydration structuring, we can, therefore, switch the solid–solid adhesion on and off, rendering the SAM into a tunable and effective electromechanical adhesion switch.

In order to explore the specific interactions involving the surface functionalization and, in particular, the influence of the headgroup and backbone structure of the benzimidazole-based SAM on the interaction with the ions, we conducted F–D measurements between benzimidazole-based layers with an amine headgroup (M1) and methoxy headgroup (M2) as well as a linear cysteamine SAM (M3) reference (also exhibiting an amine headgroup).

Figure 3a shows exemplary compression and separation curves for all investigated organic layer systems measured in 1 mM NaCl under an applied potential of ±0.2 V. Apart from much higher adhesion values measured for the M3 system, even at −0.2 V vs Ag|AgCl, the different SAMs also vary in their observed HW shift. The aromatic SAMs M1 and M2 show a clear, incompressible structure at negative polarizations at high compressive forces, whereas M3 shows a complete collapse of the hydration structure.

**Figure 3:**
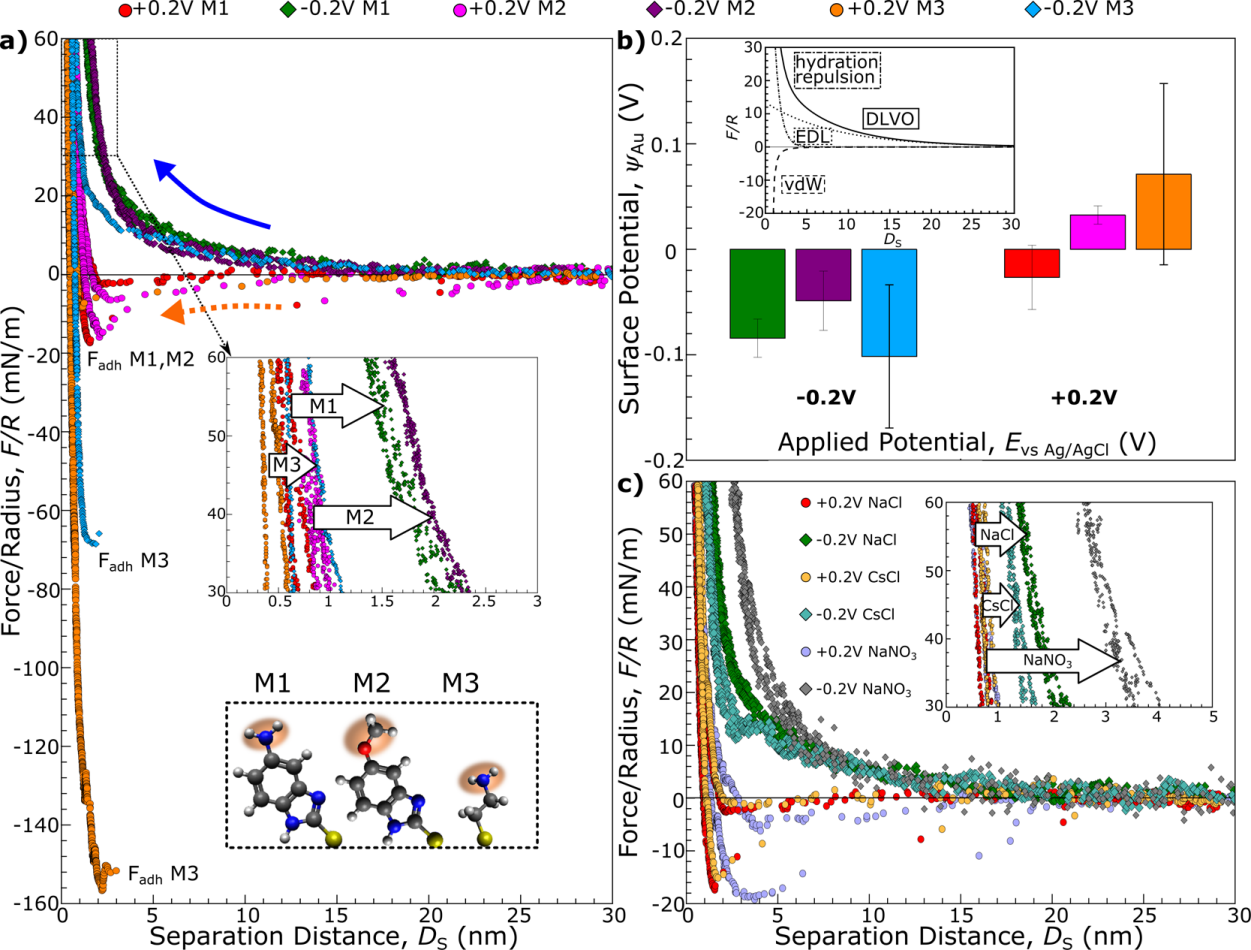
(a) F–D curves during compression and separation between mica and gold functionalized with molecules M1–M3 measured in 1 mM NaCl at +0.2 and −0.2 V external polarization (circles and diamonds, respectively). The inset graphic shows an enlarged view of the hard wall compression region, demonstrating a functionalization-dependent shift of the hard wall region linked to increased hydration structures at negative polarization. (b) Average gold surface potentials from DLVO fitting of all conducted F–D curves at applied potentials of −0.2 and +0.2 V for all 3 systems. (c) F–D profiles measured between mica and M1 functionalized gold surfaces measured in 1 mM NaCl, CsCl, and NaNO_3_, showing also ion-dependent structuring effects as highlighted by varying hard wall shifts in the inset graphic.

Notably, we observe that even though the same potential is applied to the gold substrate, adhesion and hydration decay length values can vary across F–D measurements, as shown in the statistics in Figures S4 and S5. The reason for the discrepancies between individual F–D profiles becomes clear when looking at the average surface potential at −0.2 and +0.2 V in Figure 3b, taken from the DLVO fitting of all measured F–D curves from different contact points as well as various SAM–mica pairs. In reality, the surface potential can show a significantly different picture of the electrochemical landscape at the interface from what might be suggested from the externally applied potential values. The different SAM systems with their differently charged headgroups and backbone structures mediate the surface potential in their unique way. Amine groups on aromatic M1 system for example influences the surface potential to a significant degree. The large variation in the surface potential in Figure 3c, even at the same applied potential, is likely caused by the inherent inhomogeneities of the SAM and contact environment differences between gold/SAM–mica pairs,^42^ masking general trends of adhesion and hydration within large standard deviations at the same applied potential (see Figures S4 and S5). This observation suggests that the applied potential, *E*_vs Ag|AgCl_, is not a reliable variable to correlate surface processes across different contact geometries and modifications. In order to robustly compare the different systems, we thus used the fitted surface potential ψ_Au_ of the SAM-modified gold surface instead of the applied potential *E*_vs Ag|AgCl_ as our electrochemical value to compare across systems. Using the surface potential as a measure of the actual electrochemical environment condition at the interface incorporates any charge regulation differences of the SAM as well as natural SAM and substrate defects, highlighting more clearly ion adsorption mediated trends.

To also further dive into the specific impact of different ions on the interaction mechanism, we exchanged the Na^+^ cation with Cs^+^ and the Cl^–^ anion with NO_3_^–^.

Even though the vdW radius of Cs^+^ cations is larger than that of Na^+^ cations, their measured hydration shell radius is found to be shorter. We therefore expect the hydration structure formed by Cs^+^ cations to be less ordered compared to Na^+^ cations.^10,43,44^ Figure 3c shows a comparison of ion-dependent F–D isotherms measured in the M1 system.

Even though the adhesion switch character is maintained in the CsCl system when we vary the applied potential between +0.2 and −0.2 V, the hydration repulsion is reduced, as evidenced by the smaller HW shift for the CsCl system. This observation agrees well with our interpretation that Cs^+^ cations form a less-structured near-surface hydration layer. During compression of the EDL structure, the water molecules within the hydration layer formed in the CsCl system are thus easier to compress or squeeze out than those in the NaCl system, resulting in the decreased HW shift. The role of not only cations but also anions in the organization of a stable hydration layer is further underlined by the results of nitrate-containing systems, showing an increased HW shift compared to Cl^–^ containing electrolyte.

As a means to compare in detail the hydration and adhesion properties of the different SAM and salt systems, we regrouped the data from different experiments at various external polarizations, ranging from −0.3 to +0.3 V, by surface potential (in ±10 mV width intervals) and calculated the average and standard deviation of the hydration decay length and adhesion values within each surface potential group.

Since all our SFA measurements are conducted under extremely slow surface displacement to avoid loading rate-dependent nonequilibrium forces, we converted the measured adhesion force *F*_adh_ to the surface energy γ according to the JKR model:^12,45,46^

$$F_{\mathrm{adh}}=-3\pi R\gamma$$ 1

where *R* is the SFA disk radius, *R* = 1 cm. The results of the statistical analysis are shown in Figure 4a–f and highlight the different behavior of the surface functionalizations in cooperation with different electrolytes. The HW shift of the interaction curve and increased repulsive behavior can now be directly linked to the longer range hydration repulsion and attributed to a thicker hydration layer upon cation adsorption at negative surface potentials. The influence of different ions can best be seen in the M1 system, where the rising trend in λ_hyd_ with a decreasing surface potential follows different trajectories for different ions. Notably, λ_hyd_ is smaller for CsCl than for NaCl, in line with the hydration characteristics described above. The same is valid for the M2 system, with the exception of an absent difference between different anion systems, where in M1, NO_3_^–^ shows higher λ_hyd_ values. While this observation for stronger hydration structuring for NO_3_^–^ in M1 seems contradictory to the general view of reduced ion–water interaction for anions and cation-dominated hydration effects,^12,47,48^ it underscores the crucial influence of SAM-specific ion interaction, not only between cation and SAM but also anion–SAM interaction. SFA measurements in M1 suggest NO_3_^–^, with its particular chemical structure, interacts specifically with the amine headgroup, additionally enhancing the interface hydration structure due to specific adsorption effects in M1.^49^ M3 functionalized surfaces, on the other hand, show, in general, less ion-dependent behavior when reacting to a change in surface potential.

**Figure 4:**
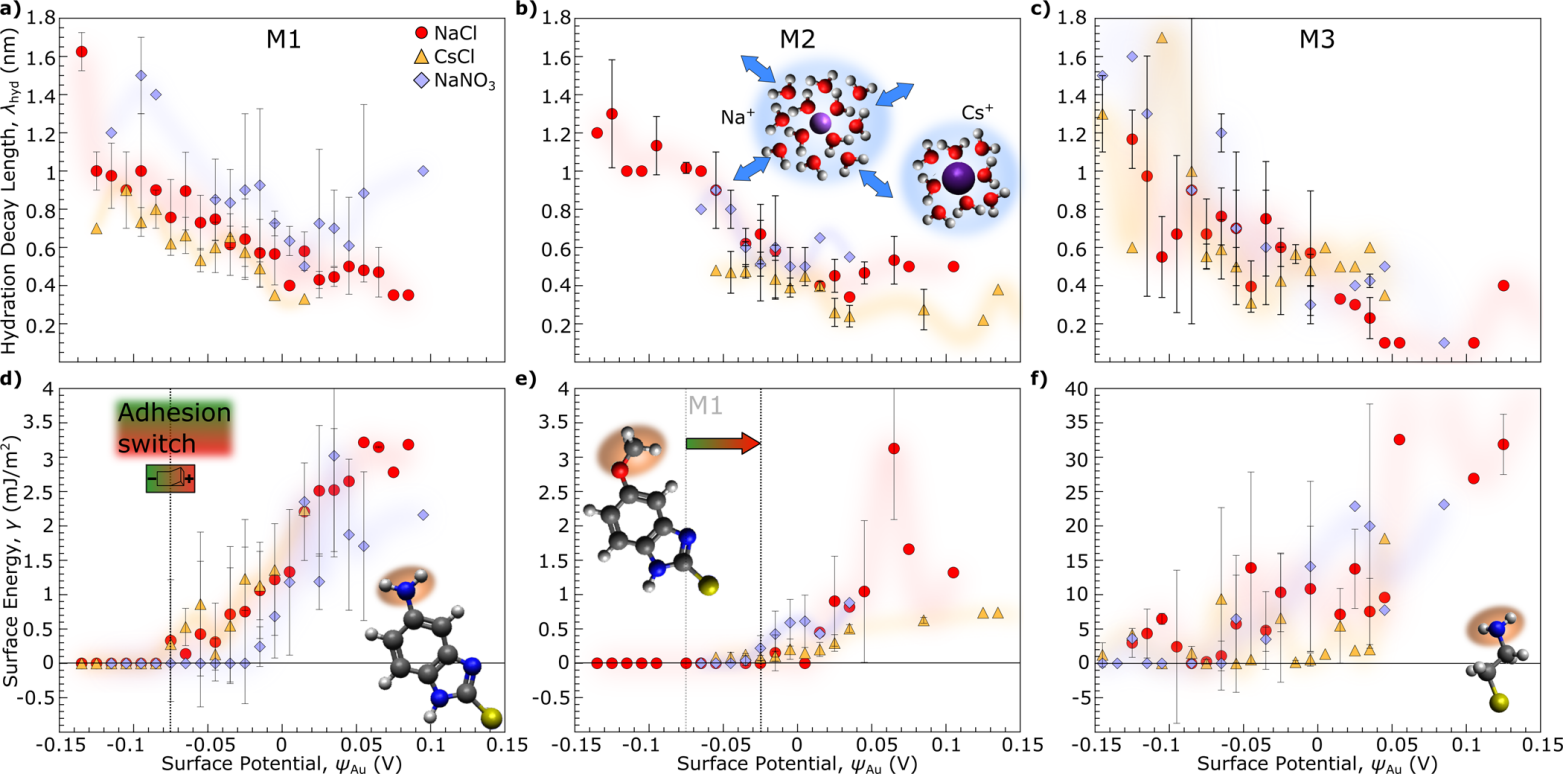
(a–c) Correlation of the fitted hydration decay length vs gold surface potential from DLVO fitting in M1 to M3 systems. Red circles, yellow triangles, and blue diamonds represent measurements in 1 mM NaCl, 1 mM CsCl, and 1 mM NaNO_3_, respectively. Inset schematics illustrate the difference in Na^+^ and Cs^+^ hydration shells, causing the hydration repulsion. (d–f) Surface energy, calculated from the measured adhesion force using a JKR model, vs gold surface potential with the same colors and symbols as above. M1 and M2 systems exhibit a clear adhesion on/off switch, albeit at different respective potentials, as highlighted in (d) and (e).

Where at negative potentials the hydration repulsion due to thicker ion and water layering is dominant, at more positive potentials, the surfaces start to interact in an attractive manner, and an onset of surface energy can be measured. When looking at the surface energy ( $\gamma =\frac{W_{\mathrm{adh}}}{2}$ ) plotted against the surface potential ψ_Au_ in Figure 4d–f, we see yet a stark difference between the SAM systems.

The M1 system shows an onset of the surface energy at potentials of −75 mV for Cl^–^ containing electrolytes. The M2 system only shows stable increasing adhesion energy at higher potentials of −25 mV. NO_3_^–^ anion-containing electrolytes show a later onset of adhesion energy compared to Cl^–^ salts in M1, more comparable to the behavior in M2, hinting again at a crucial specific interaction of anions not only in combination with Na^+^ and Cs^+^ but also together with the positively charged headgroup and subsequent anion layer structuring. While both M1 and M2 show a clear nonadhesive region at low surface potentials, M3 does not exhibit such a distinct, nonadhesive regime and, as a rule, shows much higher adhesion energies up to 20 times the magnitude compared to M1 and M2.

The results in Figure 4 clearly demonstrate the dependence of the effective interaction on the surface potential, which can be partially and indirectly modulated via the externally applied potential. Further, it shows that adhesion energy and hydration behavior are, on the one hand, specific to the type of ion adsorbing at the interface, and on the other hand, the molecular structure of the SAM layer steers the interaction mechanism with the ions as well as with the apposing mica surface.

In the cases of aromatic SAMs M1 and M2, tuning the surface potential more negatively, specific interactions of surface functional groups with mica can be shielded entirely due to increased ion adsorption-related hydration, thus following a molecular electromechanical mechanism. No adhesion energy can be measured in potential regimes below −65 mV and above λ_hyd_ > 0.85 nm for either SAM. However, with the NH_2_ headgroup containing layer, adhesive interaction can be switched on already at lower surface potentials compared to M2. Altogether, surface energy is higher for contacts involving an NH_2_–mica interaction, which may be attributed to the electrostatic attraction between positively charged amine functional groups and the negatively charged mica surface.^50^ In comparison, the MeO-terminated SAM carries no charge and therefore contributes less specific interaction, and only the surface charge from the gold substrate can interact with mica from a farther distance.

The presented trends for hydration decay length and surface energy are only evident from a combined analysis of the multiple systems and conditions over a large set of F–D curves, accumulated across multiple surface pairs, and repetitive measurements. While individual F–D profiles exhibit characteristic features for lower and higher surface potentials, such as adhesion at positive polarization and repulsion at negative polarization, variability in magnitude due to, for example, contact deformation or local differences in the monolayer, can eventually mask the general underlying statistical trends related to the surface potential. Therefore, it is necessary to obtain information from an ample set of surface pairs and measurements from different contact positions to acquire a comprehensive overview of the surface potential landscape.

Although the SFA analysis can give us a picture of the overall effect of ion adsorption and surface functionalization on interaction forces, we are not able to infer from the F–D data alone why the aromatic and linear SAMs behave so differently or how the hydration structure exactly looks like.

Therefore, to further substantiate our experimental results and hypothesis of an ion and SAM-specific modulation of hydration and adhesion, we performed molecular dynamics simulations of the gold–SAM–electrolyte interface, under positive and negative applied external electric fields in NaCl electrolyte as a representative system for the ion modulation effects. To mimic the confinement structure present in SFA experiments, a mica lattice with a negative surface charge was placed at one end of the 5 nm simulation box. Figure 5a–d shows ion density profiles as a function of distance from the gold surface in M1 and M3 SAM systems in NaCl solution.

**Figure 5:**
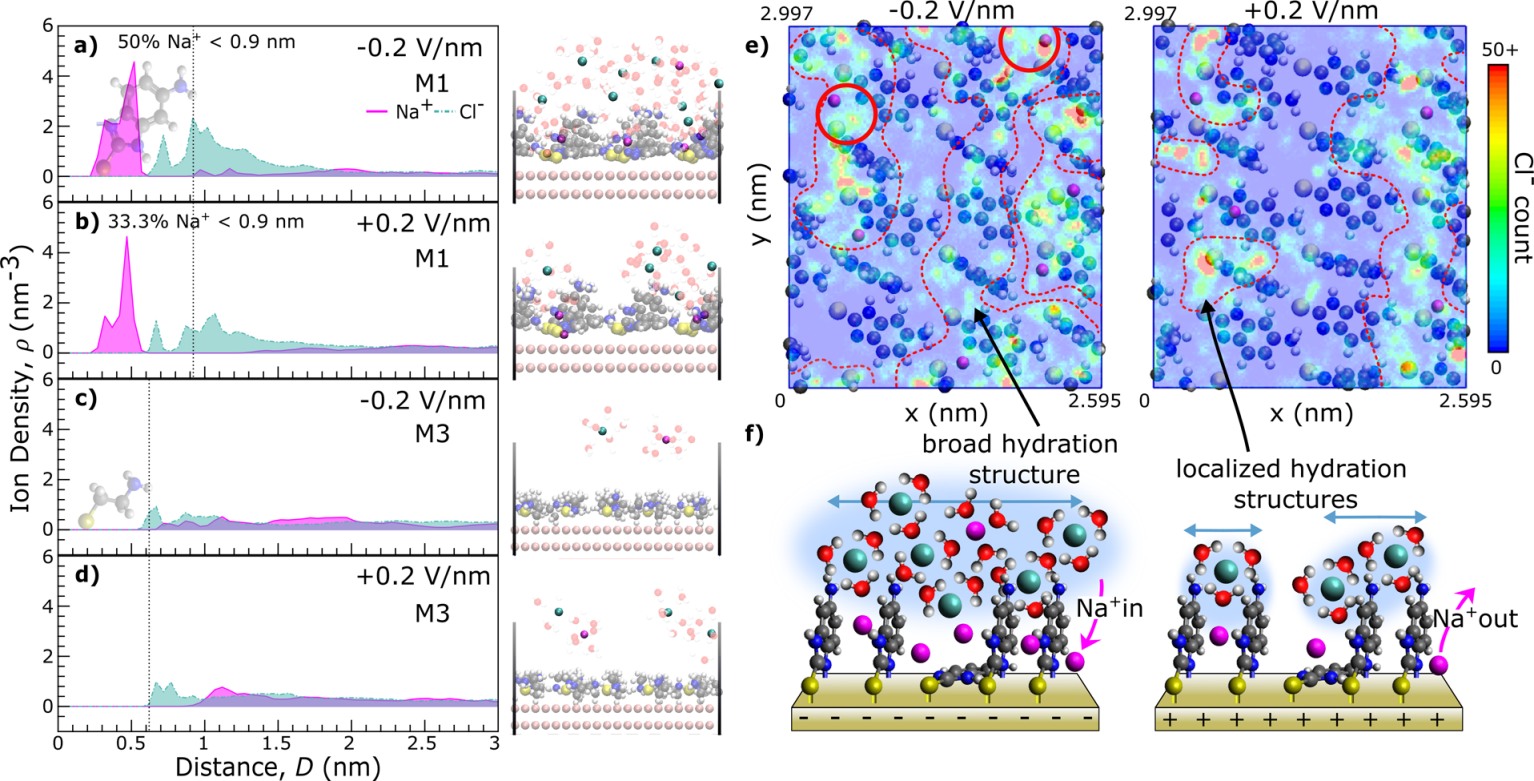
(a–d) Simulated ion density profiles of the SAM-electrolyte-mica system under applied fields of ±0.2 V/nm, with (a) and (b) displaying Na^+^ and Cl^–^ density profiles within and at the M1 layer as magenta and green lines, respectively. (c and d) The profiles for Na^+^ and Cl^–^ in the M3 system indicate no ions inside the layer. Simulation snapshots corresponding to the density profile are shown next to the panels. (e) Comparing lateral maps of the M1 SAM interface at −0.2 and +0.2 V/nm with the color code corresponding to the cumulative number of Cl^–^ at this position within a simulation time frame of 900 ns. The increased Cl^–^ density and broader hydration structure at −0.2 V/nm, in contrast to a more localized structure at +0.2 V/nm, is highlighted by dashed lines and higher Cl^–^ counts in association with increased trapped Na^+^ at −0.2 V are marked in red. (f) Illustrates the two different hydration structures.

A key finding for the M1 system is that Na^+^ ions can penetrate and remain trapped within the aromatic SAM, close to the heterocyclic ring, independent of our applied polarization. While Na^+^ preferably stays inside the aromatic layer, Cl^–^ anions accumulate around the amine headgroup. At −0.2 V/nm, contrary to what would be expected from an electrostatic perspective, a dense Cl^–^ layer forms near the M1 interface, as evidenced by the broad peak in the density profile in Figure 5a. When the field is reversed to +0.2 V/nm, some, but not all, of the trapped Na^+^ ions are expelled from the layer (reduction from 50% of total Na^+^ < 0.9 nm to 33.3%), and Cl^–^ density directly at the interface is congruently reduced, weakening the interfacial hydration structure.

The M3 system, in comparison, is not able to retain any cations within the linear molecule layer. Although Cl^–^ again stays closer to the amine headgroup, it is predominantly Na^+^ ions that react to the electric field and are able to organize closer to the SAM interface at −0.2 V/nm.

The density profile for the M1 system suggests a unique ion–ion and ion-SAM interaction, with Cl^–^ ions remaining associated with trapped Na^+^ and hence anchored close to the interface despite the repulsive applied electric field. The generally higher density of interfacial Cl^–^ for the M1 system is also seen in the simulation snapshots in Figure 5. To investigate this pattern further, we analyzed the lateral distribution of Cl^–^ across the M1 interface in Figure 5e. The heat map of Cl^–^ counts overlaid with the SAM molecules and trapped Na^+^ distribution confirms that Cl^–^ preferentially stays close to the amine headgroup in both polarizations. However, at −0.2 V/nm, the increase in Na^+^ confined within the SAM layer leads to not only an increased Cl^–^ count close to the cation position, but the anion distribution is generally more spread out laterally across the SAM compared to the +0.2 V/nm case (encircled regions in Figure 5e). This hints at the stabilizing ability of trapped Na^+^ on interfacial Cl^–^, acting against the electric field. Trapped Na^+^ can form apparent ion pairs with associated Cl^–^ in some cases and form an extended, broad ion hydration structure across the interface at −0.2 V/nm (as highlighted in Figure 5e). Both effects lead to a more rigid, incompressible ion hydration layer, as seen in the F–D measurements. In contrast, Cl^–^ at +0.2 V forms less dense and more localized structures, leading to a noncontinuous ion hydration layer. Nitrate is a more weakly hydrated, polarizable (’softer’) anion than chloride. As such, it can more readily shed part of its hydration shell and penetrate into the SAM layer,^51,52^ where it specifically associates with cationic sites and trapped Na^+^, see Figure S9. Classical treatments of hydration forces emphasize that hydration repulsion is dominated by strongly hydrated cations, while anions are considered weakly hydrated and thus not expected to contribute significantly.^12,53^ In this framework, chloride is indeed more tightly hydrated than nitrate. Our data, however, reveal that nitrate produces a stronger hydration repulsion and shift in adhesion onset in M1 (Figure 4), due to its capability of penetrating into the SAM layer and thus participating directly in interfacial structuring when paired with trapped Na^+^ and positively charged sites, increasing interfacial density and stability. This observation highlights the active role that anions, in addition to cations, play in hydration structuring and underscores that specific anion-SAM interactions can actively modulate hydration structuring at the interface.

To validate our findings of specific Na^+^ retention at negative polarization in the presence of the organic layer, we conducted XPS measurements on M1, M3, and bare electrode systems after electro-polarization in NaCl in Figure S8. Our XPS results show that Na^+^ can be discovered in the SAM systems with increased adsorption into the M1 layer without the presence of Cl, confirming specific SAM-Na interaction rather than incomplete removal of NaCl.

Based on the combined findings from SFA measurements and MD simulations, we can now present a coherent mechanism for the electrochemically induced adhesion switch in the aromatic SAM system, sketched in Figure 5f: Under negative polarization, more Na^+^ ions are stabilized between the aromatic backbone of the SAM, drawing more Cl^–^ ions closer to the SAM interface. Cl^–^ forms a dense, structured, and most importantly, extended and broader hydration layer across the interface that resists compression and forms the last incompressible layer measured as a hard wall in SFA experiments, shielding the attractive SAM-mica interaction. Under positive polarization, electrostatic repulsion causes a partial release of Na^+^ ions from the SAM, and the remaining Na^+^ cannot effectively anchor Cl^–^ ions near the interface. The loss of this stable and broad ion pair network reduces the broader and denser hydration structure to a depleted and more localized one. The depleted hydration structure thus offers less resistance toward compression, allowing the mica to come into direct contact with the SAM, resulting in strong attractive interactions. Strong specific ion–pair interaction with the aromatic SAM system as a driving force for hydration structuring is further confirmed by comparison with that of the NaNO_3_ system. Less tightly hydrated NO_3_^–^ are more readily anchored by Na^+^ inside the M1 layer and hence more difficult to compress, showing a shifted adhesion on-switch in SFA measurements, due to additional penetration of NO_3_^–^ into the SAM and further stabilization of the Na^+^–NO_3_^–^ pair inside the M1 layer, as revealed by MD simulation in Figure S9.

In contrast, the M3 SAM lacks structural features that can trap cations and hence organize hydration layers close to the interface. Consequently, ions remain diffuse and cannot electromechanically shield the amine-mica interaction as effectively during the polarization switch, resulting in poorly modulated adhesive behavior, dominated by direct electrostatic interactions of SAM and mica.

## Conclusions and Outlook

In this work, we combined electrochemical SFA measurements and MD simulations to investigate the modulation of adhesion forces between mercaptobenzimidazole with amino (M1) and methoxy (M2) end groups, and cysteamine (M3) SAM-functionalized gold and mica surfaces by means of controlling the interfacial ion and hydration structure. Our findings reveal that increasing the surface potential via external polarization reduced the hydration decay length, which governs the interaction force profile at close separations. As the hydration layer thins and breaks apart with increasing positive polarization, the resulting attractive contact is characterized by the direct interaction of the SAM with mica, a contact otherwise shielded by hydration repulsion at negative potentials. Crucially, the specific interaction of ions with the SAM and their potential-dependent structuring within the innermost EDL emerged as a key factor in defining an electromechanical adhesion switch. MD simulations revealed that aromatic SAM functionalization, through its unique cation-trapping capability at aromatic ring structures, creates a higher-density, broader Cl^–^ interfacial layer at negative potentials, facilitating an extended and more stable hydration structure that resists compression and shields SAM–mica interaction, functioning as an effective electromechanical adhesion switch. Comparatively, linear molecular systems like M3, lacking such structural motifs, exhibited less distinct interfacial co-ion structuring and correspondingly weaker modulation of adhesion and no switching behavior.

This combined experimental and computational study discloses an adhesion switch mechanism that acts without chemical reactions and underscores the critical role of the ion-SAM interaction and combined interfacial anion ordering in modulating interfacial forces for surface–surface interactions in electrolyte solutions. Our work offers a strategic framework using organic layers for leveraging ion adsorption and hydration as tunable mechanisms to control surface interactions that are heavily influenced by the EDL structure and, more specifically, the interaction of ions with interfacial modifications. This opens up potential applications in fields using targeted adhesion control, e.g., protein recognition and design of molecular grippers, and in fields like electrocatalysis and corrosion, where surface reactivity is modulated by the structure of the EDL.

## Experimental Section

### Chemicals and Materials

5-Amino-2-Mercaptobenzimidazole (purity >97%) and 2-Mercapto-5-Methoxybenzimidazole (purity >98%) were purchased from Tokio Chemical Industries for preparation of a 1 mM ethanolic solution (99% Ethanol). NaCl (99.5%, Acros), CsCl (99%, Thermo Scientific), and NaNO_3_ (99%, Thermo Scientific)­were used for salt solutions, prepared with Milli-Q water (Merck). SFA metal thin film substrates of 30–50 nm thickness were prepared with a Quorum Q300T T Plus sputter machine, where gold targets were sputtered for 5 min at 50 mA, and back-silvered mica was prepared via sputtering for 4 min with 80 mA. SFA gold surfaces (disk curvature = 1 cm) were prepared via template stripping from a mica substrate using a UV curable glue (Norland Adhesive, NO81). Back-silvered mica surfaces were prepared similarly by gluing the substrates onto the SFA quartz glass disks.

### SAM Preparation

SAMs were prepared in a 1 mM ethanol solution, and the freshly template stripped gold surfaces were immersed overnight at room temperature. After functionalization, the surfaces were rinsed with ethanol and gently dried under a N_2_ gas stream before mounting onto the SFA setup. XPS characterization of SAM thin films was carried out with a PHI instrument equipped with an Al Kα 1.5 keV source, further specified in the SI.

### Surface Forces Apparatus

In order to measure F–D isotherms and detect the intermolecular forces between SAM-functionalized gold and mica surfaces, we employed a modified SFA as described in detail in previous works.^25^ In short, an interferometric cavity between cross-cylindrically arranged semitransparent mirrors was created to measure separation distance via multiple-beam interferometry^54^ while measuring forces exerted onto the system via a highly sensitive strain gauge (ME Systems). The thin gold film functions as a substrate for thiol functionalization as well as a working electrode. A platinum wire around the quartz glass disks was used as a counter electrode, and a mini Ag|AgCl reference electrode was added to the liquid cell. Electrochemical modulation was then carried out with a CHI potentiostat without any further *iR* compensation in a potential window of −0.3 to +0.3 V (CV shown in Figure S7). The apposing surface consisted of back-silvered mica. The final system setup is sketched out in Figure 1a. During SFA measurements, the surfaces were repeatedly compressed and separated via a piezoelectric motor. The interferometric data were subsequently analyzed with the SFA Explorer Software Package using multiple matrix method to calculate the thickness of the layer system.^55^ Distance and force data were combined after alignment and thermal drift correction to obtain the F–D curves. DLVO fitting was later carried out according to the model in the SI, using a nonlinear least-squares algorithm.

### Molecular Dynamics Simulation

We conducted *NVT* molecular dynamics simulations using the open-source GROMACS 2022.5 package^56^ with a time step of 1 fs and a coupling time constant of 1 ps for the Nosé–Hoover thermostat.^57,58^ The three-site SPC/E model^59^ was used to represent water, while the OPLS-AA force field^60^ was applied for Na^+^, Cl^–^, 5-Amino-2-Mercaptobenzimidazole (M1), and Cysteamine (M3). We adopted the force field developed by Schaefer and co-workers^61^ to accurately describe NaNO_3_ in aqueous solution. The gold substrate consisted of four immobilized atomic layers, exposing the Au(111) surface in the *z*-direction.

SAMs of M1 were constructed in a 3 × 6 × 1 supercell corresponding to a 1/6 monolayer (ML) coverage, based on prior studies demonstrating the 1/6 ML structure as the most stable configuration on Cu surfaces.^29^ Due to the strong interaction between sulfur/nitrogen and gold atoms, the S and N atoms of each SH-BimH molecule were constrained to their nearest Au atoms on the surface with a fixed bond length of 3 Å. Similarly, SAMs of M3 were built in a 5 × 6 × 1 supercell corresponding to a 1/4 ML coverage, as reported by Zhang et al.^62^

To investigate the effect of interfacial confinement, a mica surface was placed on the opposite side of the simulation box from the SAM–Au interface. To determine the ion distribution at the interface, an *NVT* simulation was first performed using a 20 nm-thick water layer over 100 ns, from which the ion density profile within 5 Å of the surface was extracted. Based on this profile, a new system configuration was prepared: the gold–SAM structure and 12 NaCl ion pairs were retained, and a 5 nm-thick water layer was added above the SAM–Au surface. This water layer contained 1256 and 1268 water molecules for the M1 SAM systems under −0.2 and +0.2 V/nm electric fields, respectively, and 1441 and 1455 water molecules for the M3 SAM systems under the same conditions.

A soft Lennard-Jones wall was introduced at the top of the *z*-direction to prevent water molecules from escaping, and periodic boundary conditions were applied in the *x* and *y* directions. Long-range Coulombic interactions were computed by using a pseudo-2D Particle Mesh Ewald (PME) summation. To prevent artificial periodicity in the *z*-direction, a 15 nm vacuum layer was added above the water layer.

The simulation box for M1 systems had dimensions *a* = 2.595 nm, *b* = 2.997 nm, and *c* = 20 nm with angles α = β = γ = 90°, while for M3 systems the dimensions were *a* = 2.890 nm, *b* = 3.006 nm, and *c* = 20 nm, consistent with the Au(111) unit cell geometry. An external static electric field of ±0.2 V/nm was applied along the *z*-direction over a 5 nm span. Although the field strength and ion concentrations do not exactly match experimental conditions, they are sufficient to reproduce potential-dependent trends observed experimentally.

Simulations began with energy minimization using the steepest descent algorithm, followed by a 100 ns *NVT* pre-equilibration at 300 K. Production runs were conducted for 200 ns under *NVT* conditions at 300 K. This simulation setup enables detailed investigation of interfacial ion and water behavior, SAM structuring, and electric field effects at the SAM–Au–electrolyte interface.

## Supplementary Material



## Notes

Diffusion coefficient calculations from MD simulation and DLVO model specifications, additional reference measurements in Milli-Q water, statistical analysis, CV and XPS measurements (PDF)
