# Supplementary material for: Electrically Switchable Molecular Adhesion via Self-Assembled Monolayer-Mediated Hydration and Ion Structuring
Source: J Am Chem Soc. 2025 Nov 7;147(46):42416–25. doi: 10.1021/jacs.5c11903 (PMC12636003; doi:10.1021/jacs.5c11903)
Supplement: Supplementary file 1 [file ja5c11903_si_001.pdf]

Supplementary Information for Electrically Switchable Molecular Adhesion via SAM-Mediated Hydration and Ion Structuring

Valentina Wieser,[*,†] Yoyo Cheng-Ting Yu,[‡,¶,§] Andrea Valencia Ramirez,[‖,⊥,†] David T. Wu,[‡,§] Frank Uwe Renner,[*,‖,⊥,†,#,@] and Hsiu-Wei Cheng[*,†]

†*Department of Chemistry, National Taiwan University, Taipei 10617, Taiwan*

‡*Institute of Chemistry, Academia Sinica, Taipei 115, Taiwan*

¶*Sustainable Chemical Science and Technology, Taiwan International Graduate Program, Academia Sinica and National Taiwan University, Taipei 10617, Taiwan*

§*Department of Chemical Engineering, National Taiwan University, Taipei 10617, Taiwan*

‖*Institute for Materials Research (IMO), Hasselt University, Wetenschapspark 1, 3590 Diepenbeek, Belgium*

⊥*IMEC vzw. Division IMOMEC, 3590 Diepenbeek, Belgium*

#*Competence Center for Electrochemical Surface Technologies CEST, Viktor-Kaplan-Strasse 2, 2700 Wiener Neustadt, Austria*

@*Institute of Applied Physics, TU Wien, Wiedner Hauptstrasse 8-10/E134, 1040 Wien, Austria*

E-mail: d10223202@ntu.edu.tw; frank.renner@tuwien.ac..at; williamcheng@ntu.edu.tw

CONTENTS

1 DLVO model **2**

2 Water Diffusion Coefficients in Electrolyte Solution by Molecular Dynamics Simulation **4**

3 Supporting Figures **6**

A Fittting Table **13**

1 DLVO MODEL

The force-law according to DLVO theory predicts that in a highly compressed contact, when the separation distance D_S between two surfaces is extremely short, the attractive vdW force will dominate the overall cross-body interaction in low salt concentrations, resulting in an inevitably attractive contact.[S1] However, this model fails to explain our repulsive system during cation adsorption, making an addition of a short-range repulsion force necessary.[S2] The need for including a short range repulsive force, in the form of a steric hydration force stemming from structured water molecules around the ions and at the interface, is also evident when considering our observed HW shift and its link to a potential-dependent hydration structure change. To get a qualitative grasp on the intermolecular forces that act upon the surfaces and mathematically link the hydration features to the applied potential and adhesion, we fitted the experimental data to a DLVO model for asymmetric surfaces with a modification to include a term for an exponential hydration repulsion[S3] and charge regulation.[S4]

$$\frac{F}{R} = -\frac{A_H}{6(D-D_{vdW})^2} + \frac{2\pi\varepsilon\varepsilon_0 \cdot (2\psi_1\psi_2 e^{-\kappa(D-D_{EDL})} + [(2p_1-1)\psi_2^2 + (p_2-1)\psi_1^2] e^{-2\kappa(D-D_{EDL})})}{1-(2p_1-1)\cdot(2p_2-1)\cdot e^{-2\kappa(D-D_{EDL})}} + W_H \cdot e^{-\frac{D-D_{hyd}}{\lambda_{hyd}}} \quad (1)$$

The first term encompasses the vdW force using an effective Hamaker constant A_H for the gold-SAM-mica system of $2 \cdot 10^{-20}$ J.[S5] The second term describes the EDL force between the asymmetric surfaces where ψ_1 is the mica surface potential, fixed between -0.1 V and -0.12 V, according to mica-mica reference measurements. ψ_2 is the gold/SAM surface potential that depends on the external polarization. κ is the concentration dependent inverse Debye length and is set at 0.1 $\pm$0.02 nm^{-1} for the 1 mM monovalent salt concentrations used in this work. p1 and p2 are charge regulating parameters varying between 1=constant charge and 0=constant potential boundary conditions.[S4] The charge regulating parameter p1 for mica is set constantly at 1 whereas p2 for the modified gold surface varies between 1 for highly negative, strongly hydrated/charged surface conditions and 0 for higher surface potentials where ions are expelled from the surface. The last term describes the smeared out, exponential hydration repulsion with its pre-factor W_H (0.08 - 0.1 J/m^2) and hydration decay length λ_{hyd}.[S6,S7] D_{vdW}, D_{EDL} and D_{hyd} describe the separation distance at which the respective forces start to act. D_{vdW}, D_{EDL} and D_{hyd} are defined by the respective smallest separation distance at highest compression force measured for each first contact within a measurement series at positive external applied potential (thinnest hydration layer condition). Values are subsequently fixed for fitting of polarization switch repetitions within each set.

2 WATER DIFFUSION COEFFICIENTS IN ELECTROLYTE SOLUTION BY MOLECULAR DYNAMICS SIMULATION

We conducted NVT molecular dynamics simulations utilizing the open-source GROMACS 2022.5 package, with a 1 fs timestep, and a 1 ps coupling time constant for the Nosé–Hoover thermostat. The three-site SPC/E model was used for water, and the OPLS-AA force field was used for Na^+, Cs^+, and Cl^-. Periodic boundary conditions were enforced in the x, y and z direction. For each NaCl and CsCl system we put 16 pairs of salt and 224 water molecules in a $2\times2\times2$ nm^3 box. The simulations were initialized first by energy minimization using a steepest descent algorithm followed by a 9 ns NVT preequilibration at 300K. We then performed 10 successive 0.1 ns NVT runs at 300K, using each of these runs to sample velocity autocorrelations over the initial 10 ps interval of that run. We used the Green-Kubo expression

$$D = \frac{1}{3}\int_0^\infty < v_t \cdot v_{t+\triangle t} > d\triangle t \tag{2}$$

to evaluate the diffusion coefficient, D, in terms of the velocity autocorrelation function (VACF) of the velocity, v_t, of the water oxygen atom at time t. The angular brackets indicate an average over initial times t and water molecules. In practice, we used a value of $T_{cutoff} = 0.9$ ps for the upper limit for the integral, where the VACF had decayed to near zero.

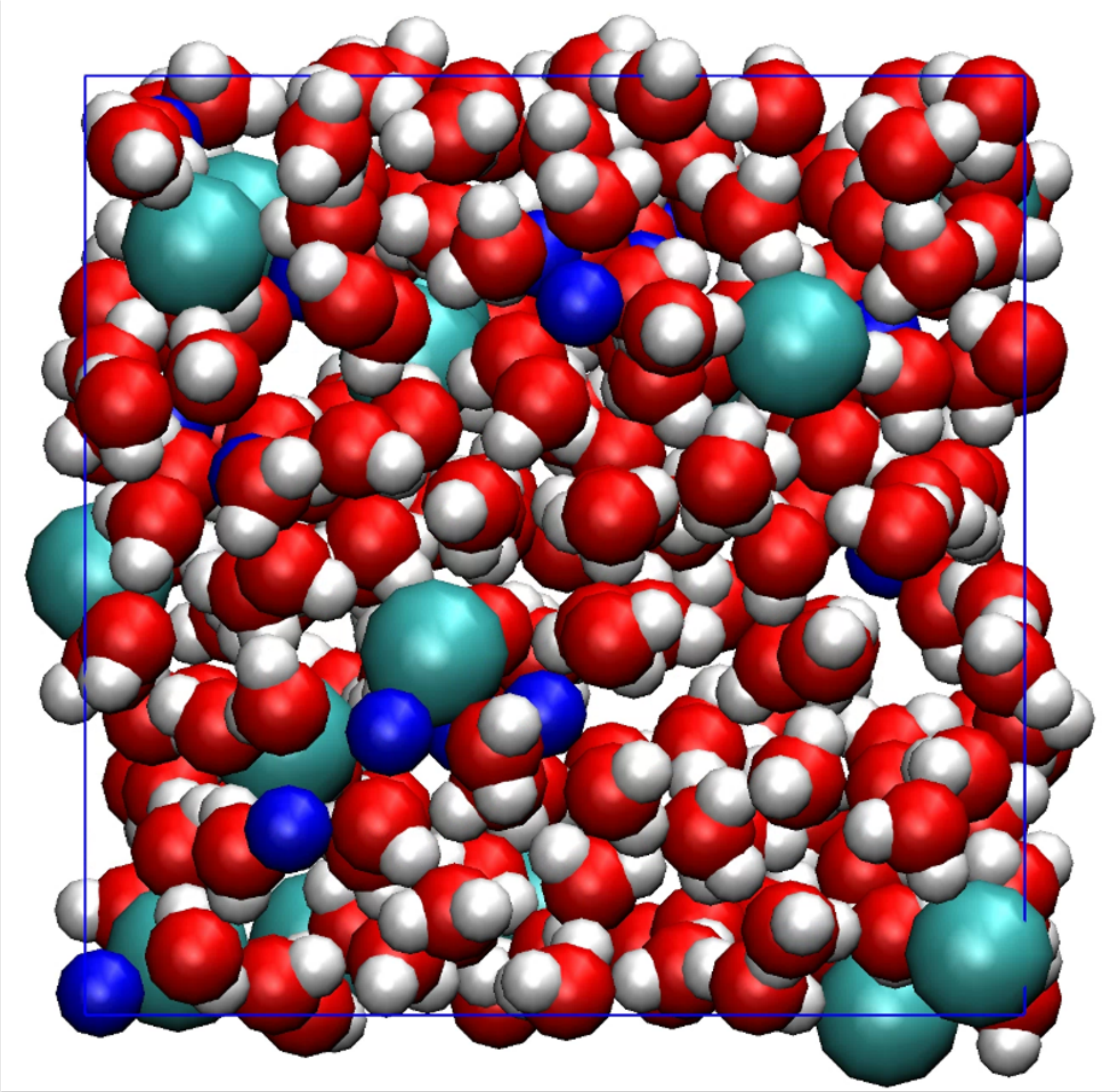

Figure S1: Simulation box for bulk diffusion coefficient calculation

Table 1: Calculated diffusion coefficients of water in a 2 x 2 x 2 nm box of ∼3 M NaCl and CsCl electrolyte solution. The value for the diffusion coefficient for pure SPC/E water is taken from Ref. S8.

	Diffusion coefficient D [10^{-5} cm^2/s]
H_2O in NaCl	2.38
H_2O in CsCl	2.74
pure H_2O	2.54

3 SUPPORTING FIGURES

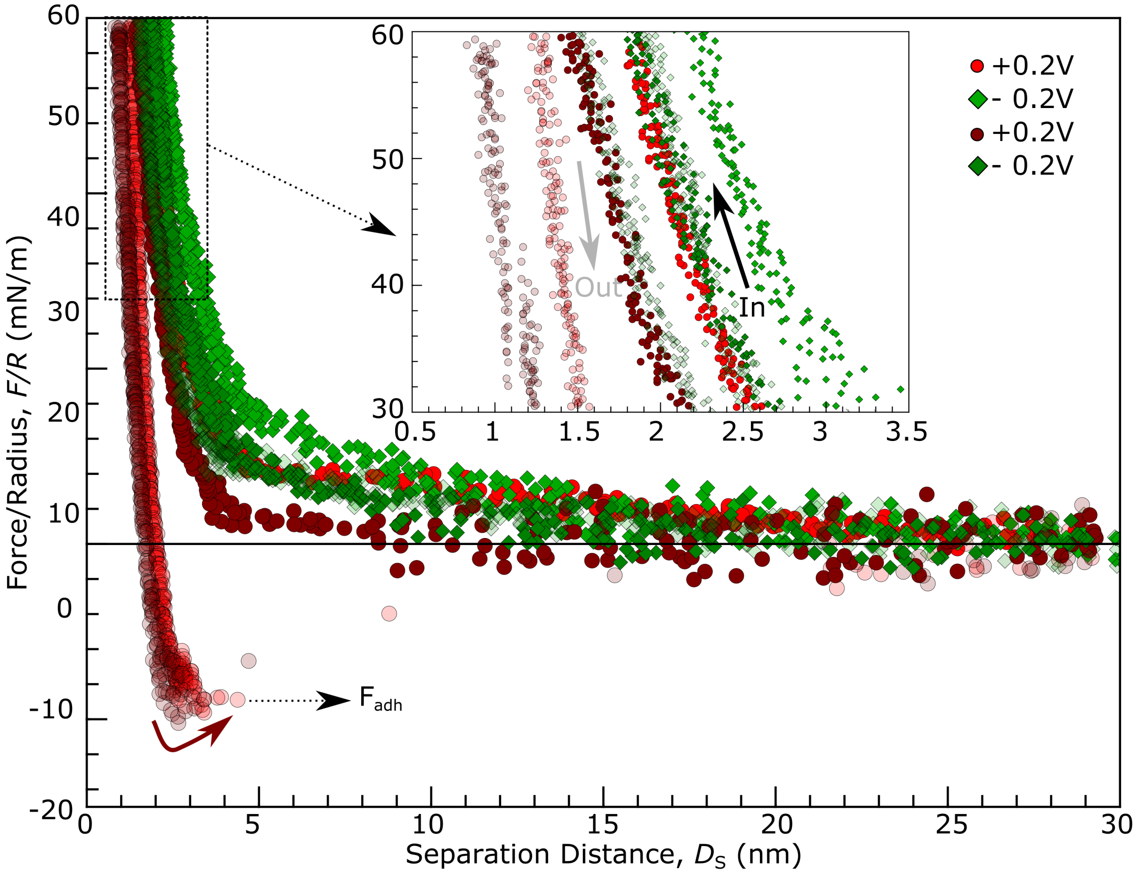

Figure S2: Four representative F-D curves out of a total of 7 repetitions between 5-Amino-2-Mercaptobenzimidazole functionalized gold and mica surface measured in MilliQ water at +0.2 V and −0.2 V in red circles and green diamonds, respectively. While the polarization switch preserves the repulsive behavior at -0.2 V and attraction at +0.2 V, no pronounced and reproducible Hard Wall shift due to prominent ion structuring can be observed. Furthermore, the hard wall region with F/R > 40 mN/m shows higher compressibility compared to cases containing confined ions.

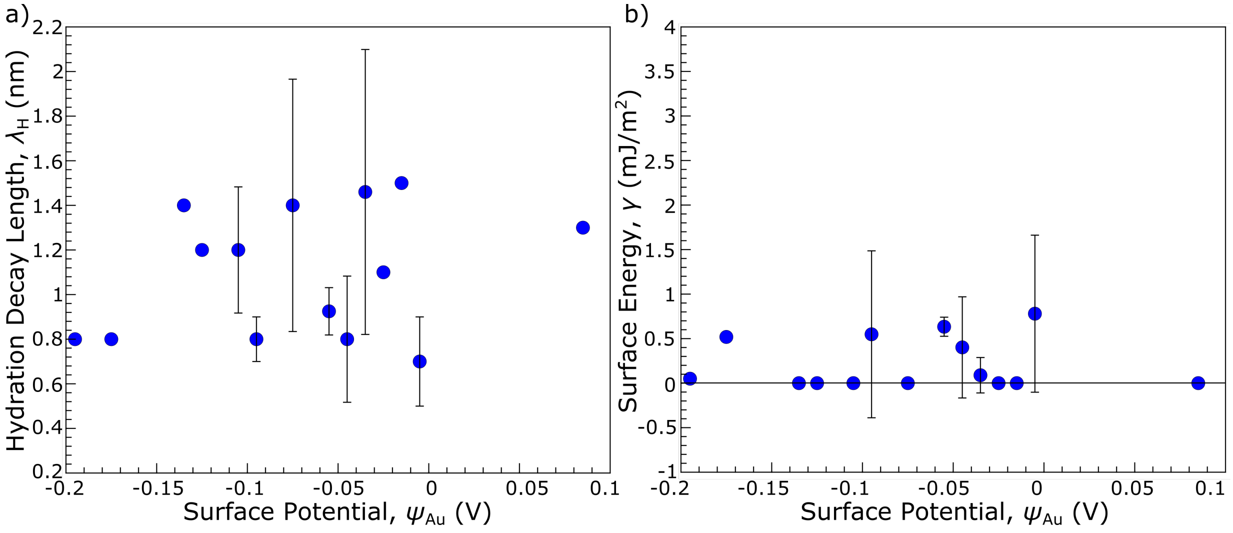

Figure S3: a) overview of fitted hydration decay length values of force-distance profiles measured between 5-Amino-2-Mercaptobenzimidazole functionalized gold and mica surface in MilliQ water, showing no clear surface potential dependent trend. b) surface energy values calculated according to JKR contact model from measured adhesion force in MilliQ water again showing no clear potential dependent trend or adhesion switch character.

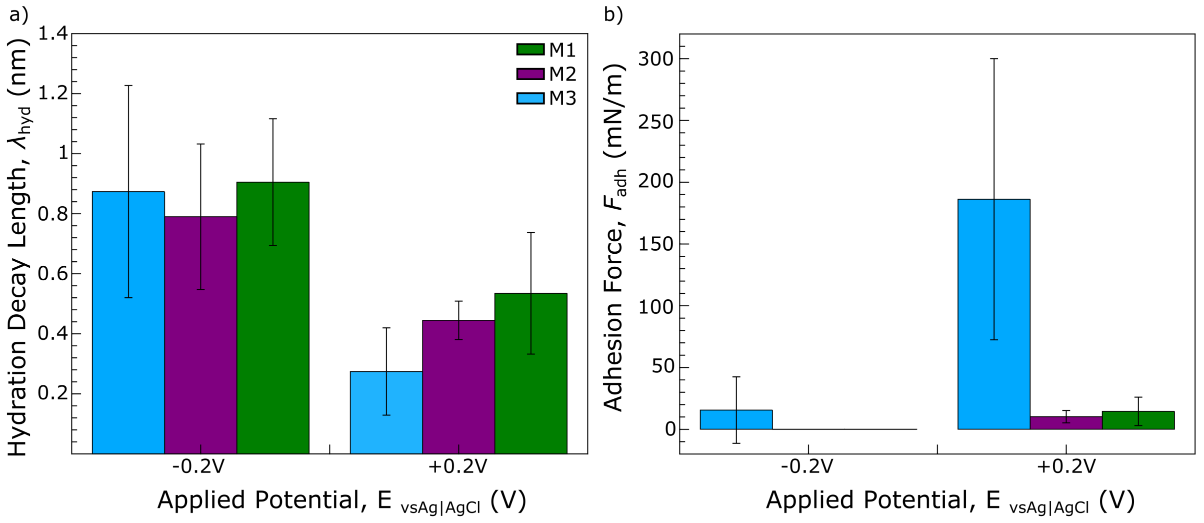

Figure S4: a) bar chart of the average hydration decay length measured during compression at ±0.2 V externally applied potential for SAM functionalization M1 (green), M2 (purple) and M3 (blue) in 1 mM NaCl. b) average measured adhesion force in mN/m for all 3 systems at ±0.2 V vs. Ag/AgCl. Average and standard deviation are calculated from all F-D measurements conducted between different surface pairs and repetitions on the same contact where −0.2 V and +0.2 V were applied. Large standard deviation is caused by discrepancy between applied potential and actual surface potential due to surface inhomogenities and surface functionalization

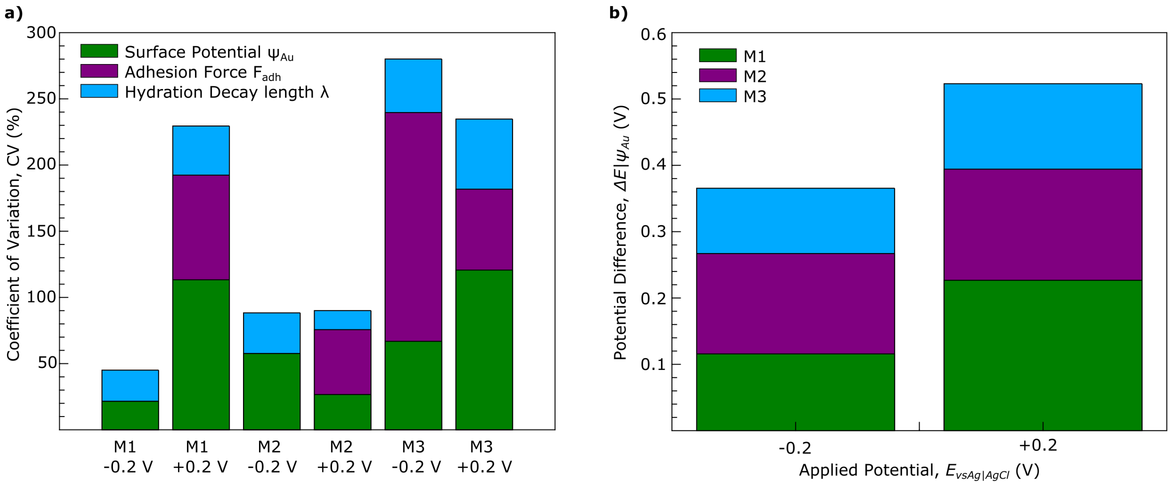

Figure S5: a) coefficient of variation of surface potential, adhesion force and hydration decay length in all 3 SAM systems in 1 mM NaCl showing large variation when referenced to the applied potential vs. Ag|AgCl b) Absolute difference between the fitted surface potential and applied potential, showing again the biggest discrepancy for the aromatic SAM terminated in an amine group at positive potentials due to anion adsorption at the positively charged headgroup

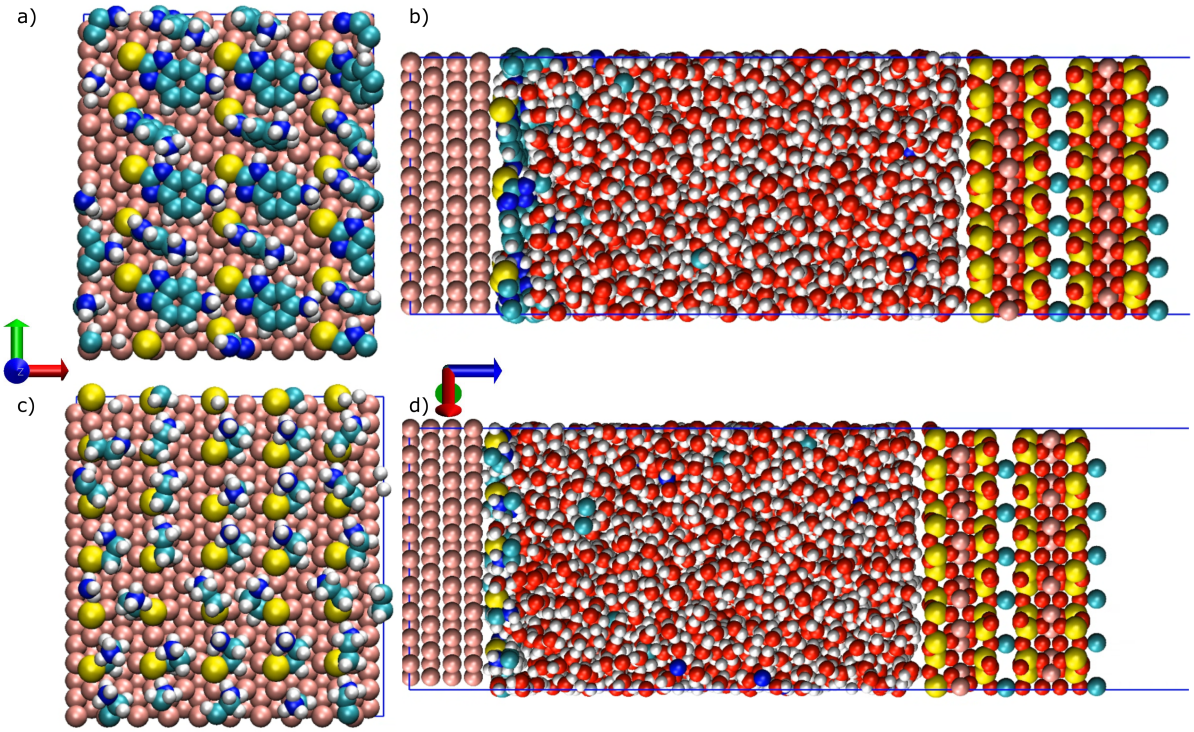

Figure S6: a) top view of the 1/6 ML monolayer structure of the 5-Amino-2-Mercaptobenzimidazole SAM functionalized gold surfaces used in the simulations b) side view of the simulation box with M1 functionalized gold on the left and mica lattice on the right of a 5 nm box filled with electrolyte. c) top view of the 1/4 Cysteamine monolayer on gold and d) showing the side view of the corresponding 5 nm simulation box with opposing mica .

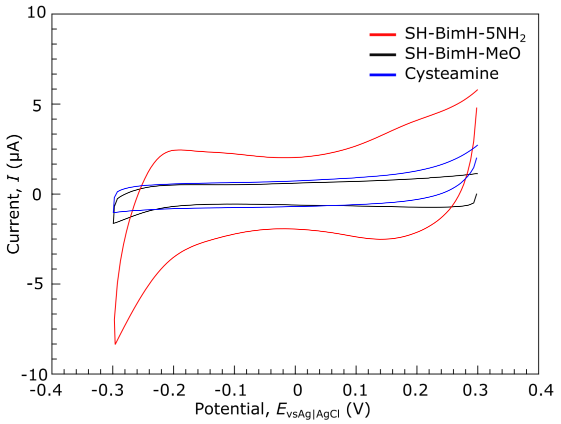

Figure S7: Cyclic Voltammetry measurements of Au electrode functionalized with 5-Amino-2-Mercaptobenzimidazol (red), 2-Mercaptio-5-Methoxybenzimidazole (black) and Cysteamine (blue) in the used potential window of −0.3 V to +0.3 V in 1 mM NaCl in an electrochemical flow cell to test for surface reactivity. Current profile in this window shows strong EDL charging behavior especially for aromatic NH_2 terminated SAM with no pronounced net redox reaction

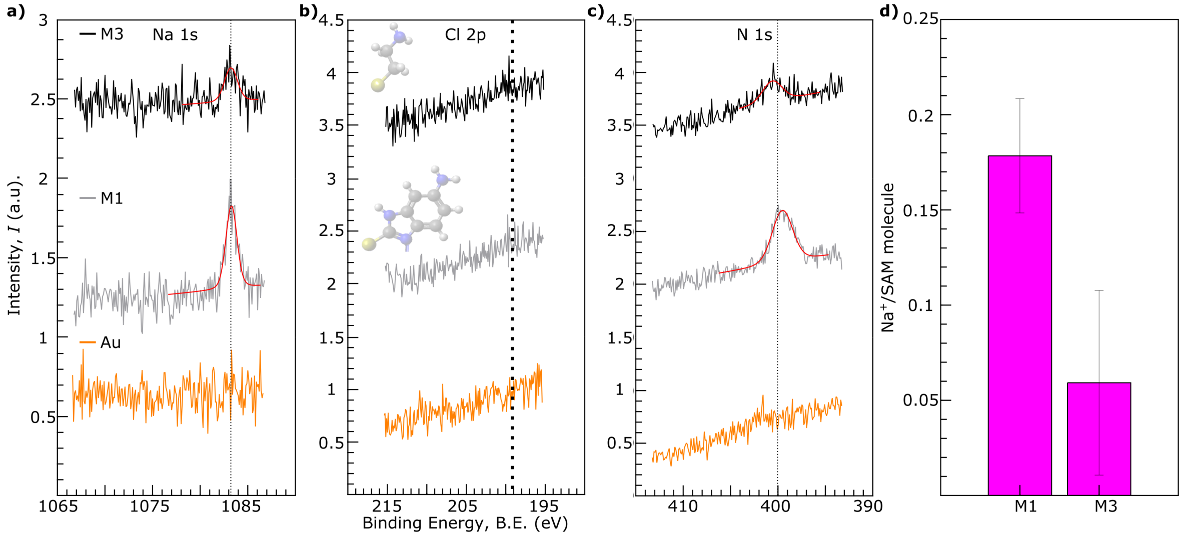

Figure S8: XPS measurements of M1 and M3 modified Au electrodes as well as bare Au reference measurements after immersion in 1 mM NaCl and 5 min polarization at -0.2 V vs. Ag|AgCl in a 3 electrode electrochemical cell with subsequent removal under recovering OCP condition (within 3 min only $\sim$ 64% of OCP was recovered) and drying under a N_2 stream. XPS measurements were conducted with an Al Kα 1.5keV source. a) Comparison of Na 1s signal after polarization, showing an increased retention of ions in the M1 system compared to the linear M3 SAM. No signal was detected for bare Au electrodes. b) shows no discernible signal for Cl 2p in either system, confirming specific adsorption of cations at negative polarization. c) shows N 1s signal used to determine organic molecule presence on the surface with higher N content in M1 system. d) bar chart comparing the Na to molecule ratio for M1 and M2 system using the Na 1s corrected peak area (RSF=8.52) and N 1s corrected peak area (RSF=1.8), confirming a higher Na content in aromatic M1 SAM system, consistent with MD simulations. Residual presence of Na in M3 system can be explained by dried Na on top of the SAM after electrochemical experiments. Most importantly, no retained Na can be measured for bare Au electrodes, underscoring the specific interaction of SAM molecules with ions

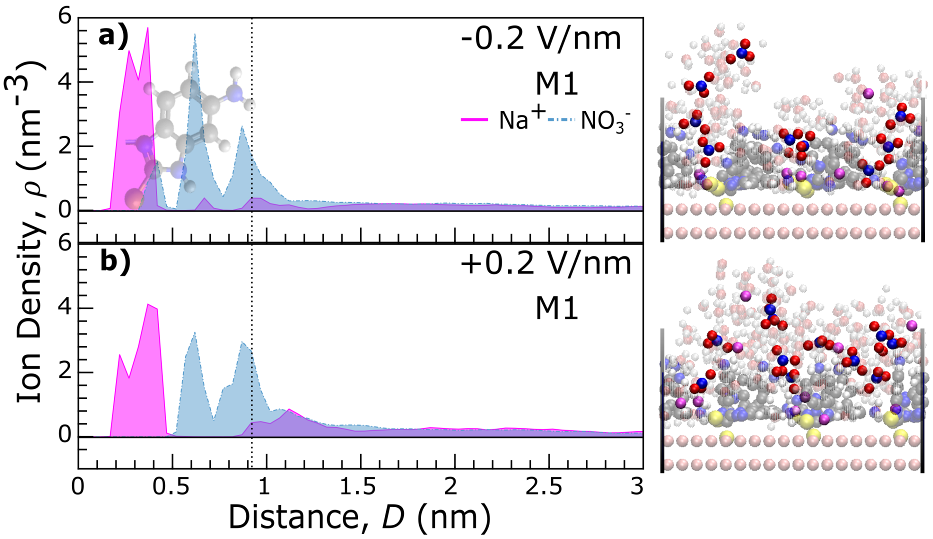

Figure S9: Simulated ion density profiles of the M1 SAM-electrolyte-mica system under applied electric fields of ± 0.2 V/nm, showing Na^+ and NO_3^- profiles as magenta and blue lines, respectively. While Na^+ again consistently stays within the SAM layer, close to the gold electrode, NO_3^- is now also able to go into the M1 layer, even at -0.2 V/nm, forming a stable ion-pair structure. Simulation snapshots of the system with interfacial ion and water are shown next to the corresponding density profiles.

References

(S1) Goldberg, R.; Chai, L.; Perkin, S.; Kampf, N.; Klein, J. Breakdown of hydration repulsion between charged surfaces in aqueous Cs+ solutions. *Physical Chemistry Chemical Physics* **2008**, *10*, 4939–4945.

(S2) Israelachvili, J. N.; Wennerstroem, H. Hydration or steric forces between amphiphilic surfaces? *Langmuir* **1990**, *6*, 873–876.

(S3) Hu, Q.; Weber, C.; Cheng, H.-W.; Renner, F. U.; Valtiner, M. Anion layering and steric hydration repulsion on positively charged surfaces in aqueous electrolytes. *ChemPhysChem* **2017**, *18*, 3056–3065.

(S4) Bilotto, P.; Lengauer, M.; Andersson, J.; Ramach, U.; Mears, L. L.; Valtiner, M. Interaction

profiles and stability of rigid and polymer-tethered lipid bilayer models at highly charged and highly adhesive contacts. *Langmuir* **2019**, *35*, 15552–15563.

(S5) Wang, J.; Yoon, R.-H. AFM forces measured between gold surfaces coated with self-assembled monolayers of 1-hexadecanethiol. *Langmuir* **2008**, *24*, 7889–7896.

(S6) Donaldson Jr, S. H.; Røyne, A.; Kristiansen, K.; Rapp, M. V.; Das, S.; Gebbie, M. A.; Lee, D. W.; Stock, P.; Valtiner, M.; Israelachvili, J. Developing a general interaction potential for hydrophobic and hydrophilic interactions. *Langmuir* **2015**, *31*, 2051–2064.

(S7) Wieser, V.; Mears, L. L.; Barker, R. D.; Cheng, H.-W.; Valtiner, M. Hydration forces dominate surface charge dependent lipid bilayer interactions under physiological conditions. *The Journal of Physical Chemistry Letters* **2021**, *12*, 9248–9252.

(S8) Vega, C.; Abascal, J. L. Simulating water with rigid non-polarizable models: a general perspective. *Physical Chemistry Chemical Physics* **2011**, *13*, 19663–19688.

A FITTTING TABLE

Solution 1 mM	FR Nr.	Appl. Potential V	A_H 10^{-20}J	D_{DLVO} nm	D_{vdW} nm	D_{hyd} nm	W_H J/m^2	κ nm^{-1}	λ nm	p_1	p_2	ψ_{Mica} V	ψ_{Au} V	Adhesion Force mN/m
M1 sample 1														
$NaNO_3$	4	OCP	2	1	1	1	0.08	0.12	1.1	1	0	-0.1	0.05	4.61
	5	0.1	2	1	1	1	0.08	0.12	1.2	1	0.2	-0.1	0.05	19.5
	6	0.1	2	1	1	1	0.08	0.12	1	1	0	-0.1	0.09	20.36
	7	-0.1	2	2	2	2	0.08	0.12	1.3	1	0.5	-0.1	-0.015	3.82
	8	-0.1	2	1	1	1	0.08	0.14	1.5	1	0.7	-0.1	-0.03	0
	9	0.2	2	1	1	1	0.08	0.12	1	1	0.7	-0.1	-0.01	5.73
	10	0.2	2	1	1	1	0.1	0.12	0.8	1	0	-0.1	0.035	18.66
	11	-0.2	2	2	2	2	0.1	0.12	1.6	1	1	-0.1	-0.1	0
	12	-0.2	2	2	2	2	0.08	0.12	1.4	1	1	-0.1	-0.1	0
	13	0.3	2	1.5	1.5	1.5	0.08	0.12	0.6	1	0.5	-0.1	0.04	6.4
	14	0.3	2	1.3	1.3	1.3	0.08	0.12	0.8	1	0	-0.1	0.04	12.61
	15	-0.3	2	1.5	1.5	1.5	0.1	0.14	1.4	1	1	-0.1	-0.09	0
	16	-0.3	2	1.5	1.5	1.5	0.1	0.12	1.2	1	1	-0.1	-0.12	01
	17	OCP	2	1	1	1	0.08	0.12	1	1	0	-0.1	0.04	8.34
	18	-0.3	2	1.5	1.5	1.5	0.1	0.14	1.7	1	1	-0.1	-0.1	0
	19	0.3	2	2	2	2	0.1	0.14	0.8	1	0.4	-0.1	-0.015	5.36
	20	0.3	2	1.5	1.5	1.5	0.1	0.14	1	1	0.5	-0.1	0.02	8.51

M1 sample 2														
$NaNO_3$	2	OCP	2	0	0	0	0.08	0.12	0.35	1	0.4	-0.1	0.0055	24.12
	4	0.1	2	0	0	0	0.08	0.12	0.5	1	0.8	-0.1	0.04	20.84
	5	-0.1	2	1	1	1	0.08	0.12	0.6	1	0.7	-0.1	-0.03	0
	6	-0.1	2	0.8	0.8	0.8	0.08	0.12	0.5	1	0.7	-0.1	-0.035	0
	7	0.2	2	0.8	0.8	0.8	0.08	0.14	0.6	1	0	-0.1	0.01	22.18
	8	0.2	2	0	0	0	0.1	0.12	0.45	1	0	-0.1	0.045	24.54
	9	-0.2	2	0	0	0	0.08	0.14	1	1	0.9	-0.1	-0.035	0
	10	-0.2	2	0	0	0	0.08	0.12	0.7	0.9	0.8	-0.1	-0.048	0
	11	0.3	2	0	0	0	0.08	0.12	0.5	1	0	-0.1	0.03	38.21
	12	0.3	2	0	0	0	0.1	0.12	0.3	1	0	-0.1	0.04	33.14
	13	-O.3	2	0	0	0	0.08	0.12	0.8	1	1	-0.1	-0.04	0
	14	-0.3	2	0	0	0	0.08	0.12	1	1	0.7	-0.1	-0.045	0
NaCl	16	0.1	2	1	1	1	0.1	0.12	0.4	1	0	-0.1	0.02	30.44
	17	0.1	2	1	1	1	0.1	0.12	0.4	1	0.4	-0.1	0.02	26.76
	19	-0.1	2	1	1	1	0.1	0.13	0.88	1	0.6	-0.1	-0.055	0
	20	0.2	2	1	1	1	0.1	0.12	0.45	1	0	-0.1	0.025	33.17
	21	0.2	2	1	1	1	0.08	0.13	0.38	1	0	-0.1	0.038	35.6
	22	-0.2	2	1	1	1	0.1	0.15	0.85	1	1	-0.1	-0.058	0
	23	-0.2	2	1	1	1	0.08	0.13	0.8	1	1	-0.1	-0.04	0
	24	0.3	2	1.2	1.2	1.2	0.1	0.12	0.48	1	0	-0.1	0.05	30.31
	25	0.3	2	1	1	1	0.1	0.12	0.38	1	0	-0.1	0.06	29.62
	26	-0.3	2	1	1	1	0.1	0.16	0.8	1	1	-0.1	-0.054	0

	27	-0.3	2	1	1	1	0.1	0.16	0.87	1	1	-0.1	-0.054	0
M1 sample 3														
$NaNO_3$	1	OCP	2	1	1	1	0.08	0.14	0.65	1	0.2	-0.1	-0.005	13.9
	2	-0.2	2	1	1	1	0.08	0.14	0.9	1	1	-0.1	-0.035	0
	3	0.2	2	1	1	1	0.08	0.14	0.7	1	0.2	-0.1	0.05	8.58
	4	-0.2	2	1	1	1	0.08	0.14	0.9	1	1	-0.1	-0.035	0
	5	0.2	2	1	1	1	0.08	0.14	0.7	1	0.2	-0.1	-0.05	6.13
	6	-0.3	2	1	1	1	0.08	0.12	0.9	1	0.9	-0.1	-0.035	0
	7	0.3	2	1	1	1	0.08	0.12	0.5	1	0	-0.1	0.015	16.06
	8	-0.3	2	1	1	1	0.08	0.11	0.8	1	1	-0.1	-0.03	0
	9	0.3	2	1	1	1	0.08	0.12	0.45	1	0.1	-0.1	0.02	13.9
	10	-0.1	2	1	1	1	0.08	0.12	0.8	1	0.9	-0.1	-0.025	0
	11	0.1	2	1	1	1	0.08	0.12	0.65	1	0.7	-0.1	-0.015	0
	12	-0.1	2	1	1	1	0.08	0.14	0.75	1	1	-0.1	-0.01	0
	13	0.1	2	1	1	1	0.08	0.14	0.65	1	0.6	-0.1	-0.015	0
	14	0.2	2	1	1	1	0.08	0.14	0.65	1	0.3	-0.1	0.002	2.73
M1 sample 4														
NaCl	18	OCP	2	1.5	1.5	1.5	0.1	0.1	0.4	1	0	-0.1	-0.08	29.14
	19	0.2	2	1.5	1.5	1.5	0.1	0.1	0.3	1	0.2	-0.15	-0.04	35.76
	20	0.2	2	1.5	1.5	1.5	0.08	0.1	0.4	1	0.2	-0.15	-0.06	35.14
	21	-0.2	2	1.5	1.5	1.5	0.08	0.1	1.1	1	1	-0.15	-0.13	0
	22	-0.2	2	1.5	1.5	1.5	0.09	0.12	0.9	1	1	-0.15	-0.11	0
	23	0.2	2	1.5	1.5	1.5	0.1	0.1	0.55	1	0.6	-0.15	-0.08	4.1

	25	0.2	2	1.5	1.5	1.5	0.08	0.1	0.35	1	0.8	-0.15	-0.06	32.47
	26	-0.2	2	1.5	1.5	1.5	0.08	0.1	1	1	1	-0.15	-0.12	0
	27	-0.2	2	1.5	1.5	1.5	0.08	0.11	0.8	1	1	-0.15	-0.105	0
	28	0.2	2	1.5	1.5	1.5	0.1	0.1	0.5	1	0.7	-0.15	-0.03	4.27
	29	0.2	2	1.5	1.5	1.5	0.08	0.1	0.35	1	0.3	-0.15	-0.03	35.49
	30	-0.2	2	1.5	1.5	1.5	0.08	0.09	0.9	1	1	-0.1	-0.13	0
	23	0.2	2	1.5	1.5	1.5	0.1	0.1	0.55	1	0.6	-0.15	-0.08	4.1
CsCl	31	OCP	2	1	1	1	0.08	0.1	0.55	1	0.5	-0.15	-0.03	16.49
	32	0.2	2	1	1	1	0.08	0.1	0.55	1	0.6	-0.15	-0.036	20.48
	33	0.2	2	1	1	1	0.08	0.1	0.52	1	0.2	-0.15	-0.055	19.11
	34	-0.2	2	1	1	1	0.08	0.1	0.9	1	1	-0.15	-0.11	0
	35	0.2	2	1	1	1	0.08	0.12	0.8	1	1	-0.15	-0.1	0
	36	0.2	2	1	1	1	0.08	0.1	0.52	1	0.2	-0.15	-0.055	19.11
	37	0.2	2	1	1	1	0.08	0.1	0.62	1	0.6	-0.15	-0.08	2.65
	38	-0.2	2	1	1	1	0.08	0.1	0.8	1	1	-0.15	-0.1	0
	39	-0.2	2	1	1	1	0.08	0.12	0.7	1	1	-0.15	-0.09	0
	40	0.2	2	1	1	1	0.08	0.1	0.6	1	0.5	-0.15	-0.07	5.92
	41	0.2	2	1	1	1	0.08	0.1	0.6	1	0.6	-0.15	-0.07	1.21
	42	-0.2	2	1	1	1	0.08	0.1	0.7	1	1	-0.15	-0.13	0
	43	-0.2	2	1	1	1	0.08	0.1	0.7	1	1	-0.15	-0.1	0
M1 sample 5														
NaCl	1	OCP	2	0.5	0.5	0.5	0.1	0.12	0.5	1	0.2	-0.12	-0.035	16.01
	2	0.2	2	0.5	0.5	0.5	0.1	0.12	0.45	1	0.2	-0.12	-0.04	13.81

	3	0.2	2	0.5	0.5	0.5	0.1	0.12	0.55	1	0.2	-0.12	-0.05	19.06
	4	0.2	2	0.5	0.5	0.5	0.1	0.12	0.35	1	0.25	-0.12	-0.031	11.65
	5	-0.2	2	0.5	0.5	0.5	0.1	0.13	0.9	1	1	-0.12	-0.065	0
	6	-0.2	2	0.5	0.5	0.5	0.1	0.13	1.1	1	1	-0.12	-0.07	0
	7	0.2	2	0.5	0.5	0.5	0.1	0.12	0.4	1	0.2	-0.12	-0.035	19.7
	8	0.2	2	1	1	1	0.1	0.12	0.35	1	0.35	-0.12	-0.005	26.52
	9	0.2	2	0.5	0.5	0.5	0.1	0.12	0.28	1	0.35	-0.12	-0.008	19.58
	10	-0.2	2	0.5	0.5	0.5	0.08	0.13	0.9	1	1	-0.12	-0.07	0
	11	-0.2	2	0.5	0.5	0.5	0.08	0.13	0.8	1	1	-0.12	-0.07	0
	16	0.2	2	1	1	1	0.1	0.12	0.35	1	0.3	-0.11	0.005	22.94
	17	-0.2	2	0.5	0.5	0.5	0.08	0.12	0.65	1	1	-0.12	-0.05	0
	18	-0.2	2	0.5	0.5	0.5	0.07	0.12	0.68	1	1.1	-0.12	-0.052	0
	19	0.2	2	0.5	0.5	0.5	0.1	0.12	0.3	1	0.45	-0.12	-0.02	17.17
CsCl	21	0.2	2	0.5	0.5	0.5	0.1	0.1	0.45	1	0.8	-0.12	-0.05	5.08
	22	0.2	2	0.5	0.5	0.5	0.1	0.12	0.5	1	0.8	-0.12	-0.05	3.52
	23	-0.2	2	0.5	0.5	0.5	0.1	0.12	0.7	1	1	-0.12	-0.09	0
	24	-0.2	2	0.5	0.5	0.5	0.08	0.12	0.6	1	1	-0.12	-0.095	0
	25	0.2	2	0.5	0.5	0.5	0.08	0.12	0.48	1	0.85	-0.12	-0.055	0
	26	0.2	2	0.5	0.5	0.5	0.08	0.12	0.45	1	0.775	-0.12	-0.048	0
	30	-0.2	2	1	1	1	0.09	0.12	0.65	1	1.1	-0.12	-0.04	0
	31	-0.2	2	1	1	1	0.1	0.1	0.65	1	1	-0.12	-0.05	0
	35	-0.2	2	0.5	0.5	0.5	0.09	0.12	0.7	1	1.3	-0.12	-0.04	0
	36	-0.2	2	0.5	0.5	0.5	0.09	0.1	0.65	1	1	-0.12	-0.05	0

	37	0.2	2	0.5	0.5	0.5	0.1	0.11	0.52	1	0.58	-0.12	-0.015	3.3
	38	0.2	2	0.5	0.5	0.5	0.1	0.1	0.6	1	0.6	-0.12	-0.06	5.31
M1 sample 6														
NaCl	12	OCP	2	0.5	0.5	0.5	0.1	0.12	0.38	1	0.6	-0.1	-0.01	15.96
	13	0.2	2	0.5	0.5	0.5	0.1	0.12	0.45	1	0	-0.1	-0.01	18.79
	14	0.2	2	0.5	0.5	0.5	0.1	0.12	0.4	1	0	-0.1	-0.02	17.49
	15	-0.2	2	0.5	0.5	0.5	0.08	0.12	0.7	1	1	-0.1	-0.1	0
	16	-0.2	2	0.5	0.5	0.5	0.08	0.12	0.9	1	1	-0.1	-0.12	0
	17	0.2	2	0.5	0.5	0.5	0.1	0.12	0.55	1	0.7	-0.1	-0.04	15.37
	18	0.2	2	0.5	0.5	0.5	0.1	0.12	0.4	1	0	-0.1	-0.02	18.95
	19	-0.2	2	0.5	0.5	0.5	0.1	0.12	0.9	1	1	-0.1	-0.1	0
CsCl	22	0.17	2	0.5	0.5	0.5	0.1	0.1	0.45	1	0.2	-0.1	-0.02	12.58
	23	OCP	2	0.5	0.5	0.5	0.1	0.1	0.45	1	0.1	-0.1	-0.018	11.71
	24	0.2	2	0.5	0.5	0.5	0.12	0.1	0.35	1	0	-0.1	-0.01	12.81
	25	0.2	2	0.5	0.5	0.5	0.12	0.1	0.38	1	0.2	-0.1	-0.015	15.98
	26	-0.2	2	0.5	0.5	0.5	0.08	0.1	0.8	1	0.9	-0.1	-0.09	0
	27	-0.2	2	0.5	0.5	0.5	0.1	0.1	0.8	1	0.9	-0.1	-0.1	0
	28	0.2	2	0.5	0.5	0.5	0.12	0.1	0.4	1	0	-0.1	-0.03	14.04
	29	0.2	2	0.5	0.5	0.5	0.12	0.1	0.33	1	0	-0.1	0.01	21.05
	30	-0.2	2	0.5	0.5	0.5	0.08	0.12	0.65	1	0.8	-0.1	-0.1	0
	31	-0.2	2	0.5	0.5	0.5	0.08	0.11	0.65	1	0.8	-0.1	-0.1	0
M1 sample 7														
NaCl	10	OCP	2	3.5	3.5	3.5	0.12	0.13	0.8	1	0.5	-0.1	-0.052	10.16

	11	0.2	2	3.5	3.5	3.5	0.12	0.12	0.55	1	0.7	-0.1	-0.045	12.85
	12	0.2	2	3.5	3.5	3.5	0.08	0.12	0.6	1	0.1	-0.1	-0.01	14.43
	13	-0.2	2	3.5	3.5	3.5	0.1	0.13	1.1	1	0.9	-0.1	-0.095	0
	14	-0.2	2	3.5	3.5	3.5	0.1	0.13	0.8	1	1	-0.1	-0.11	0
	16	0.2	2	3.5	3.5	3.5	0.08	0.12	0.6	1	0.1	-0.1	-0.02	13.28
	18	-0.2	2	3.5	3.5	3.5	0.08	0.13	0.65	1	1.2	-0.1	-0.08	0
CsCl	19	OCP	2	2.5	2.5	2.5	0.08	0.12	0.65	1	0.55	-0.1	-0.02	9.79
	20	0.2	2	2	2	2	0.1	0.12	0.65	1	0.35	-0.1	-0.03	8.06
	21	0.2	2	2	2	2	0.1	0.12	0.7	1	0.15	-0.1	-0.03	7.81
	22	-0.2	2	2	2	2	0.1	0.12	0.9	1	1	-0.1	-0.085	0
	23	-0.2	2.5	2.5	2.5	2.5	0.1	0.15	0.75	1	1.05	-0.1	-0.045	0
	24	0.2	2	2	2	2	0.1	0.12	0.7	1	0.75	-0.1	-0.04	4.65
	25	0.2	2	2	2	2	0.1	0.12	0.65	1	0.75	-0.1	-0.04	5.9
	26	-0.2	2	2.5	2.5	2.5	0.1	0.13	0.68	1	1.35	-0.1	-0.04	0
	27	-0.2	2	2.5	2.5	2.5	0.1	0.1	0.75	1	1.35	-0.1	-0.05	0
	20	0.2	2	2	2	2	0.1	0.12	0.8	1	0.6	-0.1	-0.049	8.06
M1 sample 8														
NaCl	7	OCP	2	4	4	4	0.08	0.15	0.68	1	0.6	-0.1	-0.015	0.82
	8	0.25	2	3.5	3.5	3.5	0.1	0.12	0.65	1	0.2	-0.1	-0.03	2.41
	9	0.25	2	3.5	3.5	3.5	0.08	0.12	0.58	1	0.2	-0.1	-0.035	4.8
	10	0.2	2	3.5	3.5	3.5	0.08	0.12	0.78	1	0.2	-0.1	-0.05	2
	11	0.2	2	3.5	3.5	3.5	0.08	0.12	0.78	1	0	-0.1	-0.035	2.63
	12	0.15	2	3.5	3.5	3.5	0.08	0.12	0.8	1	0.1	-0.1	-0.06	6.92

	13	0.15	2	3.5	3.5	3.5	0.08	0.12	0.8	1	0.2	-0.1	-0.04	1.59
	14	0.1	2	3.5	3.5	3.5	0.08	0.12	0.58	1	0.5	-0.1	-0.04	0
	16	0.1	2	3.5	3.5	3.5	0.08	0.12	0.5	1	0.5	-0.1	-0.035	0
	17	0.05	2	3.5	3.5	3.5	0.08	0.12	0.65	1	0.7	-0.1	-0.05	0
	18	0.05	2	3.5	3.5	3.5	0.08	0.12	0.6	1	0.5	-0.1	-0.05	0
	19	-0.05	2	3.5	3.5	3.5	0.08	0.12	1	1	0.9	-0.1	-0.067	0
	20	-0.05	2	3.5	3.5	3.5	0.08	0.12	1	1	1	-0.1	-0.06	0
	22	-0.1	2	3.5	3.5	3.5	0.08	0.12	0.7	1	1.2	-0.1	-0.035	0
	23	-0.15	2	3.5	3.5	3.5	0.08	0.12	0.68	1	1.3	-0.1	-0.045	0
	24	-0.15	2	3.5	3.5	3.5	0.08	0.12	0.78	1	1.2	-0.1	-0.04	0
	25	-0.2	2	3.5	3.5	3.5	0.08	0.09	0.78	1	1	-0.1	-0.072	0
	26	-0.2	2	3.5	3.5	3.5	0.08	0.12	0.82	1	1	-0.1	-0.05	0
	27	-0.25	2	3.5	3.5	3.5	0.08	0.14	0.82	1	1	-0.1	-0.045	0
	28	-0.25	2	3.5	3.5	3.5	0.08	0.14	0.75	1	0.9	-0.1	-0.058	0
	29	0.25	2	3.5	3.5	3.5	0.08	0.12	0.6	1	0.7	-0.1	-0.035	0
	30	0.25	2	3.5	3.5	3.5	0.08	0.12	0.55	1	0.6	-0.1	-0.025	0.28
M1 sample 9														
NaCl	1	0.17	2	5	5	5	0.1	0.1	0.6	1	0.2	-0.1	0.01	28.02
	2	0.2	2	4.2	4.2	4.2	0.08	0.1	0.6	1	0	-0.1	0.01	22.72
	3	-0.2	2	4	4	4	0.1	0.1	1.2	1	1	-0.1	-0.11	0
	4	0.3	2	4.2	4.2	4.2	0.08	0.1	0.45	1	0	-0.1	0.025	23.77
	5	0.3	2	4	4	4	0.08	0.1	0.45	1	0	-0.1	0.03	23.99
	6	0.25	2	4	4	4	0.08	0.1	0.65	1	0	-0.1	0.01	15.36

7	0.25	2	4	4	4	0.08	0.1	0.65	1	0	-0.1	0.01	17.97
8	0.2	2	4	4	4	0.08	0.1	0.65	1	0	-0.1	-0.01	12.02
9	0.15	2	4	4	4	0.08	0.1	0.8	1	0	-0.1	-0.01	12.04
10	0.15	2	3.5	3.5	3.5	0.08	0.1	0.78	1	0	-0.1	-0.005	12.17
11	0.1	2	3.5	3.5	3.5	0.08	0.1	0.8	1	0.3	-0.1	-0.015	5.6
12	0.11	2	3.5	3.5	3.5	0.08	0.1	0.8	1	0.3	-0.1	-0.015	7.24
13	0.05	2	3.5	3.5	3.5	0.08	0.1	0.9	1	0.5	-0.1	-0.03	1.62
14	0.05	2	3.5	3.5	3.5	0.08	0.1	0.9	1	0.5	-0.1	-0.03	1.21
15	-0.05	2	4	4	4	0.1	0.1	0.8	1	0.8	-0.1	-0.06	0
16	-0.05	2	4	4	4	0.08	0.12	0.75	1	0.8	-0.1	-0.058	0
17	-0.1	2	4	4	4	0.08	0.1	0.9	1	0.9	-0.1	-0.08	0
18	-0.1	2	4	4	4	0.08	0.1	1	1	1	-0.1	-0.08	0
20	-0.15	2	4	4	4	0.08	0.1	0.9	1	1	-0.1	-0.08	0
21	-0.2	2	4	4	4	0.08	0.1	0.9	1	1	-0.1	-0.08	0
22	-0.25	2	4	4	4	0.08	0.1	0.9	1	1	-0.1	-0.085	0
23	-0.25	2	4	4	4	0.08	0.1	0.9	1	1	-0.1	-0.085	0
24	-0.3	2	4	4	4	0.08	0.1	0.8	1	1	-0.1	-0.08	0
25	-0.3	2	4	4	4	0.08	0.12	0.75	1	1.2	-0.1	-0.06	0
26	0.2	2	3.5	3.5	3.5	0.07	0.1	0.9	1	0.6	-0.1	-0.05	1.69
27	0.2	2	2.5	2.5	2.5	0.07	0.1	1.3	1	0.3	-0.1	-0.07	1.3
28	-0.2	2	4	4	4	0.08	0.12	0.75	1	1	-0.1	-0.065	0
29	-0.2	2	3	3	3	0.08	0.1	1	1	1.5	-0.1	-0.08	0
32	0.2	2	2	2	2	0.08	0.1	0.7	1	0.4	-0.1	-0.04	3.19

	33	0.2	2	2	2	2	0.1	0.1	0.75	1	0.4	-0.1	-0.05	3.19
	34	-0.2	1.5	1.5	1.5	1.5	0.1	0.1	1.4	1	1	-0.1	-0.1	0
	35	-0.2	2.5	2.5	2.5	2.5	0.1	0.1	1.6	1	1	-0.1	-0.14	0
M1 sample 10														
NaCl	1	0.14	2	2.5	2.5	2.5	0.1	0.1	0.5	1	0.2	-0.1	0.02	19.51
	2	0.2	2	2.5	2.5	2.5	0.1	0.1	0.5	1	0.1	-0.1	0.04	24.97
	3	-0.2	2	2.5	2.5	2.5	0.08	0.12	0.88	1	1	-0.1	-0.05	0
	4	0.2	2	2.5	2.5	2.5	0.1	0.1	0.5	1	0.2	-0.1	0.03	18.4
	5	-0.2	2	2.5	2.5	2.5	0.08	0.1	0.8	1	1	-0.1	-0.05	0
	6	0.3	2	2.5	2.5	2.5	0.1	0.1	0.35	1	0.1	-0.1	0.08	30
	7	-0.3	2	2.5	2.5	2.5	0.08	0.1	0.9	1	1	-0.1	-0.07	0
	8	-0.3	2	2.5	2.5	2.5	0.1	0.1	0.35	1	0.3	-0.1	0.07	26.21
	9	0.25	2	2.5	2.5	2.5	0.1	0.1	0.45	1	0.3	-0.1	0.03	17.08
	10	0.25	2	2.5	2.5	2.5	0.1	0.1	0.52	1	0.3	-0.1	-0.01	14.8
	11	0.15	2	2.5	2.5	2.5	0.1	0.1	0.62	1	0.5	-0.1	-0.02	5.39
	12	0.15	2	2.5	2.5	2.5	0.1	0.1	0.62	1	0.3	-0.1	-0.01	5.72
	13	0.1	2	2.5	2.5	2.5	0.1	0.1	0.72	1	0.3	-0.1	-0.02	5.12
	14	0.1	2	2.5	2.5	2.5	0.1	0.1	0.7	1	0.3	-0.1	-0.02	6.02
	15	-0.1	2	2.5	2.5	2.5	0.08	0.1	0.82	1	0.9	-0.1	-0.055	0
	16	-0.1	2	2.5	2.5	2.5	0.08	0.12	0.82	1	0.8	-0.1	-0.045	0
	17	-0.15	2	2.5	2.5	2.5	0.08	0.12	0.82	1	0.8	-0.1	-0.055	0
	18	-0.15	2	2.5	2.5	2.5	0.08	0.1	0.9	1	0.8	-0.1	-0.05	0
	19	-0.25	2	2.5	2.5	2.5	0.08	0.1	0.9	1	0.8	-0.1	-0.055	0

	20	-0.25	2	2.5	2.5	2.5	0.08	0.12	0.8	1	1	-0.1	-0.045	0
	21	-0.3	2	2.5	2.5	2.5	0.08	0.14	0.8	1	1	-0.1	-0.045	0
	22	-0.3	2	2.5	2.5	2.5	0.08	0.14	0.8	1	1	-0.1	-0.055	0
	23	0.2	2	2.5	2.5	2.5	0.08	0.12	0.8	1	1	-0.1	-0.01	5.92
M1 sample 11														
NaCl	1	0.13	2	3.5	3.5	3.5	0.1	0.1	0.45	1	0.5	-0.1	0.005	12.54
	2	0.2	2	1.5	1.5	1.5	0.1	0.12	0.42	1	0.2	-0.1	-0.005	6.7
	3	-0.2	2	1.5	1.5	1.5	0.1	0.12	0.7	1	1	-0.1	-0.065	0
	4	0.2	2	1.5	1.5	1.5	0.1	0.12	0.38	1	0.6	-0.1	-0.02	4.28
	5	-0.2	2	1.5	1.5	1.5	0.1	0.12	0.9	1	1	-0.1	-0.065	0
	6	0.3	2	1.5	1.5	1.5	0.1	0.12	0.38	1	0.4	-0.1	0.01	10.25
	7	-0.3	2	1.5	1.5	1.5	0.1	0.14	0.7	1	1	-0.1	-0.05	0
	8	0.3	2	1.5	1.5	1.5	0.1	0.12	0.38	1	0.3	-0.1	0.02	8.37
	9	-0.3	2	1.5	1.5	1.5	0.08	0.12	0.7	1	1	-0.1	-0.08	0
	10	0.25	2	2	2	2	0.1	0.12	0.58	1	0.4	-0.1	-0.005	4.44
	11	-0.25	2	1	1	1	0.1	0.12	0.6	1	1	-0.1	-0.07	0
	12	0.25	2	2	2	2	0.1	0.12	0.58	1	0.4	-0.1	-0.005	4.56
	13	-0.25	2	1	1	1	0.08	0.14	0.55	1	1	-0.1	-0.04	0
	16	0.3	2	2	2	2	0.1	0.12	0.6	1	0.4	-0.1	-0.01	3.44
	18	0.2	2	1	1	1	0.1	0.12	0.45	1	0.3	-0.1	-0.03	2.89
	19	0.15	2	1	1	1	0.1	0.12	0.65	1	0.4	-0.1	-0.06	1.47
	20	0.155	2	1	1	1	0.1	0.1	0.55	1	0.4	-0.1	-0.35	0.5
M1 sample 12														

NaCl	1	0.13	2	5	5	5	0.1	0.12	0.75	1	1	-0.1	-0.01	27.69
	2	0.3	2	4.5	4.5	4.5	0.1	0.12	0.56	1	0	-0.1	0.06	29.69
	3	-0.3	2	4	4	4	0.1	0.12	1.7	1	1.1	-0.1	-0.14	0
	4	0.3	2	4.5	4.5	4.5	0.1	0.12	0.6	1	0.2	-0.1	0.01	28.32
	5	-0.3	2	4	4	4	0.1	0.1	1.7	1	0.6	-0.1	-0.14	0
	6	0.25	2	4	4	4	0.1	0.12	0.8	1	0.3	-0.1	-0.04	13.92
	7	-0.25	2	4	4	4	0.1	0.1	1.5	1	1	-0.1	-0.14	0
	8	0.25	2	4	4	4	0.1	0.12	0.65	1	0.3	-0.1	-0.04	9.28
	9	-0.25	2	4	4	4	0.08	0.1	1.2	1	0.8	-0.1	-0.12	0
	10	0.2	2	4	4	4	0.1	0.12	0.7	1	1	-0.1	-0.06	0
	11	0.3	2	4	4	4	0.1	0.12	0.7	1	1	-0.1	-0.03	5.79
	12	0.2	2	4	4	4	0.1	0.1	0.7	1	1	-0.1	-0.06	0
	14	0.2	2	4	4	4	0.1	0.14	0.5	1	0.5	-0.1	-0.01	4.1
	15	0.2	2	4	4	4	0.1	0.14	0.4	1	0.7	-0.1	-0.01	5.34
	16	-0.2	2	4	4	4	0.08	0.1	0.8	1	1	-0.1	-0.12	0
	17	0.2	2	4	4	4	0.08	0.14	0.6	1	0.9	-0.1	-0.06	0
	19	0.25	2	3	3	3	0.08	0.1	0.58	1	0.9	-0.1	-0.06	2.2
M2 sample 1														
$NaNO_3$	1	OCP	2	1	1	1	0.1	0.12	0.4	1	0.2	-0.1	0.005	3.22
	2	0.1	2	1.5	1.5	1.5	0.1	0.12	0.4	1	0.1	-0.1	-0.01	7.87
	3	0.1	2	1.5	1.5	1.5	0.1	0.12	0.4	1	0.1	-0.1	-0.015	7.15
	4	-0.1	2	1.5	1.5	1.5	0.1	0.14	0.6	1	0.4	-0.1	-0.035	1.5
	5	-0.1	2	1.5	1.5	1.5	0.1	0.12	0.	1	0.6	-0.1	-0.045	0

	8	0.2	2	1.5	1.5	1.5	0.1	0.12	0.5	1	0	-0.1	0.005	8.3
	9	0.2	2	1.5	1.5	1.5	0.1	0.12	0.55	1	0	-0.1	0.03	8.33
	10	-0.2	2	1.5	1.5	1.5	0.1	0.12	0.9	1	1	-0.1	-0.06	0
	11	-0.2	2	1.5	1.5	1.5	0.1	0.12	0.8	1	1	-0.1	-0.04	0
	12	0.3	2	1.5	1.5	1.5	0.1	0.12	0.5	1	0.1	-0.1	-0.01	3.32
	13	0.3	2	1.5	1.5	1.5	0.1	0.12	0.65	1	0.1	-0.1	0.01	4.01
	14	-0.3	2	1.5	1.5	1.5	0.1	0.14	1	1	1	-0.1	-0.02	0
	15	-0.3	2	1.5	1.5	1.5	0.1	0.1	0.8	1	0.9	-0.1	-0.07	0
NaCl	18	0.1	2	1.5	1.5	1.5	0.1	0.12	0.5	1	0	-0.1	0.1	12.44
	19	0.1	2	1.5	1.5	1.5	0.1	0.12	0.5	1	0.9	-0.1	0.07	15.68
	20	-0.1	2	1.5	1.5	1.5	0.1	0.12	0.8	1	0.9	-0.1	-0.055	0
	21	-0.1	2	1.5	1.5	1.5	0.1	0.12	0.8	1	0.9	-0.1	-0.06	0
	23	0.2	2	1.5	1.5	1.5	0.1	0.12	0.5	1	0	-0.1	0.04	14.67
	24	0.2	2	1.5	1.5	1.5	0.1	0.12	0.5	1	0	-0.1	0.04	14.47
	25	-0.2	2	1.5	1.5	1.5	0.1	0.12	1	1	1	-0.1	-0.08	0
	26	-0.2	2	1.5	1.5	1.5	0.1	0.14	1	1	1	-0.1	-0.065	0
	27	0.3	2	1.5	1.5	1.5	0.1	0.12	0.6	1	0.5	-0.1	-0.02	5.27
	28	0.3	2	1.5	1.5	1.5	0.1	0.12	0.5	1	0	-0.1	0.06	17.8
	29	-0.3	2	1.5	1.5	1.5	0.1	0.12	1	1	1	-0.1	-0.08	0
	30	-0.3	2	1.5	1.5	1.5	0.1	0.12	1.2	1	1	-0.1	-0.14	0
	31	0.3	2	1.5	1.5	1.5	0.1	0.12	0.6	1	0.1	-0.1	0.06	18.45
M2 sample 2														
$NaNO_3$	4	OCP	2	1.5	1.5	1.5	0.1	0.12	0.5	1	0	-0.1	-0.02	3.1

	5	0.1	2	1.5	1.5	1.5	0.1	0.12	0.5	1	0.3	-0.1	-0.04	1.38
	6	0.2	2	1.5	1.5	1.5	0.1	0.12	0.5	1	0	-0.1	-0.03	3.87
	7	0.2	2	1.5	1.5	1.5	0.1	0.12	0.45	1	0.2	-0.1	-0.03	1.34
	8	-0.2	2	1.5	1.5	1.5	0.1	0.12	0.7	1	1	-0.1	-0.05	0
	9	0.1	2	1.5	1.5	1.5	0.1	0.12	0.7	1	1	-0.1	-0.035	0
	10	0.3	2	1.5	1.5	1.5	0.1	0.12	0.5	1	0	-0.1	-0.02	5.760
	11	0.3	2	1.5	1.5	1.5	0.1	0.12	0.5	1	0	-0.1	-0.025	3.02
	12	-0.3	2	1.5	1.5	1.5	0.1	0.12	0.7	1	1	-0.1	-0.04	0
	13	-0.3	2	1.5	1.5	1.5	0.1	0.13	0.6	1	1	-0.1	-0.038	0
	15	0.1	2	1.5	1.5	1.5	0.1	0.12	0.6	1	0.3	-0.1	-0.03	0
	17	-0.1	2	1.5	1.5	1.5	0.1	0.12	0.6	1	0.8	-0.1	-0.04	0
	18	-0.1	2	1.5	1.5	1.5	0.1	0.12	0.6	1	0.7	-0.1	-0.04	0
NaCl	19	OCP	2	1.5	1.5	1.5	0.08	0.1	0.4	1	0.3	-0.1	0.015	4.22
	21	0.1	2	1.5	1.5	1.5	0.08	0.1	0.45	1	0.3	-0.1	0.02	2.2
	23	-0.1	2	1.5	1.5	1.5	0.07	0.1	0.55	1	1	-0.1	-0.015	0
	25	0.2	2	1.5	1.5	1.5	0.08	0.1	0.4	1	0.25	-0.1	0.025	6.05
	26	0.2	2	1.5	1.5	1.5	0.08	0.1	0.38	1	0.3	-0.1	0.025	5.73
	27	-0.2	2	1.5	1.5	1.5	0.07	0.12	0.58	1	1	-0.1	-0.025	0
	28	-0.2	2	1.5	1.5	1.5	0.07	0.12	0.58	1	1	-0.1	-0.025	0
	29	0.3	2	1.5	1.5	1.5	0.08	0.1	0.38	1	0.3	-0.1	0.02	5.46
	30	0.3	2	1.5	1.5	1.5	0.08	0.1	0.34	1	0.35	-0.1	0.03	7.69
	31	-0.3	2	1.5	1.5	1.5	0.07	0.12	0.62	1	1	-0.1	-0.032	0
	32	-0.3	2	1.5	1.5	1.5	0.07	0.13	0.61	1	1	-0.1	-0.031	0

M2 sample 3														
NaCl	2	OCP	2	1.5	1.5	1.5	0.1	0.12	1.05	1	1	-0.1	-0.075	0
	3	0.2	2	1.5	1.5	1.5	0.08	0.1	0.6	1	0	-0.1	0.02	19.04
	4	-0.2	2	1.5	1.5	1.5	0.08	0.12	1	1	1	-0.1	-0.07	0
	5	0.2	2	1.5	1.5	1.5	0.08	0.1	0.68	1	0	-0.1	0.005	13.54
	6	-0.2	2	1.5	1.5	1.5	0.1	0.12	1	1	1	-0.1	-0.07	0
	7	0.1	2	1.5	1.5	1.5	0.08	0.1	0.4	1	-0.6	-0.1	0.04	31.41
	8	OCP	2	1.5	1.5	1.5	0.08	0.1	1	1	1	-0.1	-0.07	0
	9	0.3	2	1.5	1.5	1.5	0.08	0.1	0.35	1	0	-0.1	0.065	31.22
	10	-0.3	2	1.5	1.5	1.5	0.08	0.1	1.5	1	1	-0.1	-0.125	0
	11	0.3	2	1.5	1.5	1.5	0.08	0.1	0.5	1	0	-0.1	0.02	12.6
	12	-0.3	2	1.5	1.5	1.5	0.08	0.12	0.85	1	1	-0.1	-0.025	0
	13	0.1	2	1.5	1.5	1.5	0.08	0.12	0.6	1	0.9	-0.1	-0.015	0
	14	-0.1	2	1.5	1.5	1.5	0.08	0.1	1	1	0.9	-0.1	-0.11	0
M2 sample 4														
NaCl	3	-0.2	2	2.5	2.5	2.5	0.08	0.12	1.2	1	1	-0.1	-0.06	0
	1	OCP	2	2.5	2.5	2.5	0.1	0.12	0.7	1	-0.2	-0.1	0.065	31.4
	9	-0.2	2	2.5	2.5	2.5	0.08	0.12	1.1	1	1	-0.1	-0.095	0
	2	0.2	2	2	2	2	0.1	0.12	0.6	1	-0.1	-0.1	0.065	42.9
	7	0.2	2	2	2	2	0.1	0.1	1	1	0.9	-0.1	-0.12	0
	4	0.3	2	2.5	2.5	2.5	0.1	0.12	0.45	1	0	-0.1	0.065	35
	5	-0.3	2	2.5	2.5	2.5	0.1	0.1	1.1	1	1	-0.1	-0.13	0
	6	0.1	2	2.5	2.5	2.5	0.08	0.1	1.3	1	1	-0.1	-0.095	0

	8	-0.1	2	2.5	2.5	2.5	0.08	0.1	1	1	0	-0.1	-0.1	0
M2 sample 5														
CsCl	1	OCP	2	1.5	1.5	1.5	0.08	0.1	0.38	1	0	-0.1	0.13	65.54
	2	0.2	2	1.5	1.5	1.5	0.08	0.1	0.35	1	0	-0.1	0.08	58.23
	3	-0.2	2	1.5	1.5	1.5	0.08	0.1	0.6	1	0.9	-0.1	-0.028	5.41
	4	0.1	2	1.5	1.5	1.5	0.08	0.1	0.55	1	0.2	-0.1	-0.03	10.25
	5	-0.1	2	1.9	1.9	1.9	0.08	0.1	0.5	1	0.3	-0.1	-0.04	12.2
	6	0.25	2	1.9	1.9	1.9	0.08	0.1	0.15	1	0	-0.1	0.02	41.91
	8	0.3	2	1.8	1.8	1.8	0.08	0.1	0.1	1	0	-0.1	0.15	59.22
	9	-0.3	2	1.8	1.8	1.8	0.08	0.1	0.45	1	0.65	-0.1	-0.05	6.06
	10	0.2	2	1.8	1.8	1.8	0.08	0.1	0.5	1	0.8	-0.1	-0.058	24.54
	11	-0.2	2	1.8	1.8	1.8	0.08	0.1	0.45	1	0.3	-0.1	-0.04	10.22
	13	0.2	2	1	1	1	0.08	0.1	0.3	1	0	-0.1	-0.05	15.18
	15	-0.2	2	1.8	1.8	1.8	0.08	0.12	0.48	1	0.9	-0.1	-0.015	1.06
	16	0.2	2	1.8	1.8	1.8	0.08	0.12	0.4	1	0.3	-0.1	-0.012	9.74
	17	-0.1	2	1.8	1.8	1.8	0.08	0.12	0.4	1	0.7	-0.1	-0.018	6.46
	18	0.1	2	1.7	1.7	1.7	0.08	0.12	0.3	1	0.3	-0.1	-0.012	21.26
	20	0.3	2	1.5	1.5	1.5	0.08	0.12	0.2	1	0	-0.1	0.03	48.39
M2 sample 6														
CsCl	7	0.2	2	0.7	0.7	0.7	0.08	0.12	0.2	1	0	-0.1	0.085	52.83
	8	-0.2	2	0.7	0.7	0.7	0.08	0.12	0.45	1	0.32	-0.1	-0.028	7.07
	9	0.1	2	0.7	0.7	0.7	0.08	0.12	0.45	1	0.4	-0.1	0.005	13.33
	10	-0.1	2	0.7	0.7	0.7	0.08	0.12	0.6	1	0.9	-0.1	-0.045	13.72

	11	0.3	2	0.7	0.7	0.7	0.08	0.12	0.22	1	0	-0.1	0.12	65.33
	12	-0.25	2	0.7	0.7	0.7	0.08	0.12	0.42	1	0.35	-0.1	-0.005	8.92
	13	0.05	2	0.7	0.7	0.7	0.08	0.12	0.4	1	0.3	-0.1	0.01	12.66
	14	-0.05	2	0.7	0.7	0.7	0.08	0.12	0.38	1	0.2	-0.1	0.012	18.13
	15	-0.2	2	0.7	0.7	0.7	0.08	0.12	0.38	1	0.45	-0.1	-0.015	10.14
	16	0.2	2	0.8	0.8	0.8	0.08	0.12	0.35	1	0.2	-0.1	-0.01	27.5
	17	-0.3	2	0.8	0.8	0.8	0.08	0.1	0.48	1	0.8	-0.1	-0.054	7.63
	18	0.2	2	1.6	1.6	1.6	0.08	0.12	0.38	1	0	-0.1	-0.012	15.66
	19	-0.2	2	1.8	1.8	1.8	0.08	0.12	1.1	1	0.5	-0.1	-0.03	4.44
	20	0.1	2	1.8	1.8	1.8	0.08	0.12	0.3	1	0.55	-0.1	-0.025	14.17
	21	-0.1	2	1.8	1.8	1.8	0.08	0.12	0.4	1	0.5	-0.1	-0.022	8.44
	22	-0.3 2		1.8	1.8	1.8	0.08	0.12	0.4	1	0.5	-0.1	-0.025	3.47
	23	0.3	2	1.6	1.6	1.6	0.08	0.12	0.28	1	0	-0.1	0.03	41.31
M2 sample 7														
CsCl	5	0.2	2	1	1	1	0.08	0.12	0.38	1	0	-0.1	0.018	30.46
	6	-0.2	2	1	1	1	0.08	0.12	0.62	1	0.5	-0.1	-0.02	2.25
	7	0.1	2	1	1	1	0.08	0.12	0.58	1	0.3	-0.1	-0.025	7.2
	8	-0.1	2	1	1	1	0.08	0.12	0.58	1	0.1	-0.1	-0.025	5.51
	9	0.3	2	1	1	1	0.08	0.12	0.3	1	0	-0.1	0.02	23.39
	10	-0.25	2	1	1	1	0.08	0.12	0.46	1	0.4	-0.1	-0.025	4.35
	11	0.05	2	1	1	1	0.08	0.1	0.42	1	0.1	-0.1	-0.03	6.09
	12	-0.05	2	1	1	1	0.08	0.1	0.4	1	0.1	-0.1	-0.02	6.17
	13	0.2	2	1	1	1	0.08	0.12	0.29	1	0	-0.1	0.026	22.17

	14	-0.2	2	1	1	1	0.08	0.12	0.5	1	0.4	-0.1	-0.048	3.05
	15	-0.2	2	1	1	1	0.08	0.12	0.5	1	0.7	-0.1	-0.045	0.439
	16	0.2	2	1	1	1	0.08	0.12	0.55	1	0.3	-0.1	-0.015	9.32
	18	0.1	2	1	1	1	0.08	0.12	0.43	1	0.2	-0.1	0.01	9.98
	20	0.3	2	1	1	1	0.08	0.12	0.3	1	0	-0.1	0.022	17.3
M3 sample 1														
NaCl	2	0.2	2	3	3	3	0.08	0.12	0.1	1	0	-0.1	0.1	253.59
	6	0.2	2	3	3	3	0.08	0.12	0.2	1	0	-0.1	0.037	151.63
	7	-0.2	2	3	3	3	0.08	0.12	0.55	1	1	-0.1	-0.1	68.96
	8	0.2	2	3	3	3	0.08	0.12	0.1	1	0	-0.12	0.24	322.08
	9	-0.2	2	3	3	3	0.08	0.12	0.7	1	1	-0.1	-0.11	66.31
	10	0.1	2	2.5	2.5	2.5	0.08	0.12	0.3	1	0	-0.12	-0.046	223.91
	11	-0.1	2	2.5	2.5	2.5	0.08	0.12	1	1	1	-0.12	-0.038	104.22
	12	0.05	2	2.5	2.5	2.5	0.08	0.12	0.2	1	0.4	-0.12	-0.01	165.42
	13	-0.05	2	2	2	2	0.08	0.12	0.6	1	1	-0.12	-0.03	135.55
	14	0.3	2	2	2	2	0.08	0.12	0.1	1	0	-0.12	0.155	402.93
	15	-0.25	2	2.5	2.5	2.5	0.08	0.12	0.6	1	1	-0.12	-0.12	79.55
	17	-0.2	2	3	3	3	0.08	0.12	1.7	1	1	-0.1	-0.12	24.91
	18	0.1	2	2.5	2.5	2.5	0.08	0.15	0.5	1	0.7	-0.12	-0.06	162.6
	16	0.2	2	2	2	2	0.08	0.12	0.4	1	0	-0.12	0.125	329.56
	19	-0.1	2	2.5	2.5	2.5	0.08	0.15	1	1	1	-0.12	-0.13	49.92
	20	0.3	2	2	2	2	0.08	0.12	0.1	1	0	-0.12	0.055	306.89
	21	-0.25	2	2	2	2	0.08	0.12	1.3	1	1	-0.12	-0.13	23.36

	22	0.05	2	2	2	2	0.08	0.12	1.1	1	1	-0.12	-0.1	137.28
	23	0.2	2	2	2	2	0.08	0.12	0.4	1	0	-0.12	0.129	271.09
	24	-0.2	2	3	3	3	0.08	0.12	1.2	1	1	-0.1	-0.13	11.01
CsCl	25	0.2	2	2.5	2.5	2.5	0.08	0.12	0.48	1	0	-0.12	-0.069	176.95
	26	-0.2	2	2.5	2.5	2.5	0.08	0.15	1.7	1	1	-0.12	-0.11	0
	27	0.1	2	3	3	3	0.08	0.12	0.6	1	1	-0.12	-0.13	39.26
	28	-0.1	2	3	3	3	0.08	0.12	1	1	1	-0.12	-0.16	0
	29	0.3	2	2.5	2.5	2.5	0.08	0.12	0.35	1	0	-0.12	0.04	171.32
	30	-0.25	2	2.5	2.5	2.5	0.08	0.15	1.4	1	1	-0.12	-0.145	0
	31	0.05	2	2.5	2.5	2.5	0.08	0.12	1.1	1	1	-0.12	-0.15	23.43
	32	-0.05	2	3	3	3	0.08	0.12	1.5	1	1	-0.12	-0.09	7.71
	33	0.2	2	2.5	2.5	2.5	0.08	0.12	0.3	1	0.3	-0.12	-0.0225	124.43
$NaNO_3$	35	0.2	2	3	3	3	0.08	0.12	0.1	1	0	-0.12	0.089	217.9
	36	-0.2	2	4	4	4	0.08	0.12	1.7	1	1	-0.12	-0.16	0
	37	0.1	2	4	4	4	0.08	0.12	1.6	1	1	-0.12	-0.13	33.98
	38	-0.1	2	3.5	3.5	3.5	0.08	0.12	1.1	1	1	-0.12	-0.22	0
	39	0.3	2	3	3	3	0.08	0.12	0.4	1	0.1	-0.12	0.02	192.08
	40	-0.25	2	4	4	4	0.08	0.12	2.3	1	1	-0.12	-0.11	0
	41	0.05	2	4	4	4	0.08	0.12	1.3	1	1	-0.12	-0.12	0
	42	-0.05	2	4	4	4	0.08	0.12	1.5	1	1	-0.12	-0.15	0
	43	0.2	2	3	3	3	0.08	0.12	0.4	1	0.5	-0.12	-0.06	119.97
	44	-0.2	2	3.5	3.5	3.5	0.08	0.12	1.9	1	1	-0.12	-0.14	0
M3 sample 2														

$NaNO_3$	3	-0.2	2	4	4	4	0.08	0.12	0.9	1	1	-0.12	-0.04	13.79
	4	0.2	2	3	3	3	0.08	0.12	0.45	1	0.3	-0.12	0.031	306.67
	5	-0.1	2	4	4	4	0.08	0.12	0.6	1	1	-0.12	-0.06	62.99
	6	0.1	2	4	4	4	0.08	0.12	0.4	1	0.2	-0.12	-0.01	215.53
	7	-0.25	2	4	4	4	0.08	0.12	0.7	1	1	-0.12	-0.04	42.25
NaCl	11	0.2	2	4	4	4	0.08	0.12	0.4	1	0.1	-0.12	-0.01	180.53
	12	-0.2	2	4	4	4	0.08	0.12	0.9	1	0.7	-0.12	-0.07	0
	13	0.1	2	4	4	4	0.08	0.12	0.5	1	0.55	-0.12	-0.03	59.25
	14	-0.1	2	4	4	4	0.08	0.1	0.54	1	0.9	-0.12	-0.075	4.39
	15	0.25	2	4	4	4	0.08	0.12	0.3	1	0.2	-0.12	0.023	168.07
	16	-0.25	2	4	4	4	0.08	0.15	0.7	1	1	-0.12	-0.055	0
	17	-0.2	2	4	4	4	0.08	0.12	0.9	1	1	-0.12	-0.01	0
	18	0.15	2	3.5	3.5	3.5	0.08	0.12	0.78	1	0.15	-0.12	-0.01	63.04
	19	0.25	2	4	4	4	0.08	0.12	0.3	1	0.2	-0.12	0.022	91.22
	20	-0.25	2	4	4	4	0.08	0.12	0.6	1	0.9	-0.12	-0.035	0
CsCl	21	0.2	2	4.5	4.5	4.5	0.08	0.12	0.4	1	0.8	-0.12	-0.085	20.67
	22	-0.2	2	4.5	4.5	4.5	0.08	0.12	0.6	1	0.7	-0.12	-0.08	0
	23	0.2	2	3.8	3.8	3.8	0.08	0.12	0.4	1	0.3	-0.12	-0.005	20.94
	24	-0.1	2	3.8	3.8	3.8	0.08	0.12	0.48	1	0.5	-0.12	-0.075	0
	25	0.1	2	4	4	4	0.08	0.12	0.6	1	0.9	-0.12	0.005	12.83
	26	-0.1	2	4	4	4	0.08	0.12	0.4	1	0.4	-0.12	-0.059	0
	27	0.25	2	3.2	3.2	3.2	0.08	0.12	0.5	1	0.1	-0.12	0.015	87.83
	28	-0.25	2	3.8	3.8	3.8	0.08	0.12	0.5	1	0.6	-0.12	-0.06	0

M3 sample 3														
$NaNO_3$	7	0.1	2	0	0	0	0.08	0.12	0.3	1	0.3	-0.12	-0.033	42.97
	8	-0.1	2	0.5	0.5	0.5	0.08	0.12	1.3	1	1	-0.12	-0.07	0
	9	0.2	2	0	0	0	0.08	0.12	0.5	1	0.1	-0.12	0.04	73.01
	10	-0.2	2	0	0	0	0.08	0.12	1.1	1	1	-0.12	-0.07	0
	11	0.3	2	0	0	0	0.08	0.12	0.2	1	0	-0.12	-0.01	50.25
	12	-0.25	2	0	0	0	0.08	0.12	0.9	1	1	-0.12	-0.09	0
	13	0.2	2	0	0	0	0.08	0.12	0.4	1	0	-0.12	0.03	69.78
	14	-0.2	2	0	0	0	0.08	0.12	1.2	1	1	-0.12	-0.055	0
NaCl	15	0.1	2	0	0	0	0.08	0.12	0.33	1	0	-0.12	0.015	67.17
	16	-0.1	2	0	0	0	0.08	0.1	0.9	1	0.6	-0.12	-0.09	0
	17	0.2	2	0	0	0	0.08	0.12	0.4	1	0.1	-0.12	-0.04	76.28
	18	-0.2	2	0	0	0	0.08	0.12	0.8	1	1	-0.12	-0.06	0
	19	0.3	2	0	0	0	0.08	0.12	0.1	1	0	-0.12	0.04	90.34
	20	-0.25	2	0	0	0	0.08	0.12	1	1	1	-0.12	-0.035	0
	21	0.2	2	0	0	0	0.08	0.12	0.1	1	0	-0.12	0.03	44.03
	22	-0.2	2	0	0	0	0.08	0.12	0.8	1	1	-0.12	-0.07	0
	23	-0.2	2	0	0	0	0.08	0.1	0.8	1	0.8	-0.12	-0.072	0
	24	0.25	2	0	0	0	0.08	0.12	0.28	1	0	-0.12	0.03	60.06
CsCl	25	0.1	2	1	1	1	0.08	0.12	0.5	1	0	-0.12	0.01	15.4
	26	-0.1	2	1	1	1	0.08	0.12	0.7	1	0.1	-0.12	-0.07	0
	27	0.2	2	1	1	1	0.08	0.12	0.5	1	0.1	-0.12	0.02	17.7
	28	-0.2	2	1	1	1	0.08	0.12	0.6	1	1	-0.12	-0.01	0

	30	-0.2	2	1	1	1	0.08	0.12	0.5	1	1	-0.12	-0.01	0
	31	0.2	2	1	1	1	0.08	0.12	0.6	1	0.1	-0.12	0.03	19.19
	32	-0.25	2	1	1	1	0.08	0.12	0.5	1	1	-0.12	-0.01	0
M3 sample 4														
NaCl	1	OCP	2	0	0	0	0.08	0.1	0.4	1	0.6	-0.1	-0.11	55.69
	3	OCP	2	0.5	0.5	0.5	0.08	0.1	0.33	1	0	-0.1	0.03	52.48
	4	0.05	2	0.5	0.5	0.5	0.08	0.1	0.55	1	0.4	-0.1	-0.065	40.82
	7	0.2	2	0.5	0.5	0.5	0.08	0.12	0.37	1	0	-0.1	0.03	47.23
	8	-0.2	2	0.5	0.5	0.5	0.08	0.11	0.63	1	0.6	-0.1	-0.095	0
	9	0.1	2	0.5	0.5	0.5	0.08	0.1	0.49	1	0.5	-0.1	-0.045	38.011
	10	-0.1	2	0.5	0.5	0.5	0.08	0.12	0.8	1	1	-0.1	-0.062	0
	11	0.3	2	0	0	0	0.08	0.12	0.62	1	0.7	-0.1	-0.12	18.44
	14	-0.2	2	0	0	0	0.08	0.1	0.9	1	1	-0.1	-0.28	0
	15	0.05	2	1	1	1	0.08	0.12	0.4	1	0.6	-0.1	-0.23	0
CsCl	17	OCP	2	0	0	0	0.08	0.12	0.53	1	0.1	-0.1	-0.02	3.39
	18	0.2	2	0	0	0	0.08	0.12	0.4	1	0.3	-0.1	-0.005	5.43
	19	-0.2	2	0	0	0	0.08	0.12	0.6	1	1	-0.1	-0.018	0
	20	0.1	2	0	0	0	0.08	0.1	0.55	1	1	-0.1	-0.028	0
	21	-0.1	2	0	0	0	0.08	0.12	0.58	1	1	-0.1	-0.072	0
	22	0.3	2	0	0	0	0.08	0.12	0.31	1	0.9	-0.1	-0.05	5.26
